# Development of a SNP Panel for Geographic Assignment and Population Monitoring of Jaguars (*Panthera onca*)

**DOI:** 10.1002/ece3.71465

**Published:** 2025-05-22

**Authors:** Gabriele Zenato Lazzari, Henrique Vieira Figueiró, Caroline Charão Sartor, Emiliano Donadio, Sebastián Di Martino, Hope M. Draheim, Eduardo Eizirik

**Affiliations:** ^1^ Laboratório de Biologia Genômica e Molecular, Escola de Ciências da Saúde e da Vida Pontifícia Universidade Católica do Rio Grande do Sul (PUCRS) Porto Alegre RS Brazil; ^2^ Instituto Tecnológico Vale—Desenvolvimento Sustentável (ITV) Belém Pará Brazil; ^3^ Wildlife Conservation Research Unit, Recanati‐Kaplan Centre University of Oxford Tubney UK; ^4^ Fundación Rewilding Argentina Buenos Aires Argentina; ^5^ National Fish and Wildlife Forensic Laboratory United States Fish and Wildlife Service Ashland Oregon USA; ^6^ Instituto Pró‐Carnívoros Atibaia SP Brazil

**Keywords:** conservation genomics, genetic management, individual identification, parentage, sexing, wildlife forensics, genômica da conservação, manejo genético, identificação individual, sexagem, parentesco

## Abstract

The jaguar ( *Panthera onca* ) is an iconic top predator that is threatened by habitat loss and fragmentation, along with an emerging expansion of poaching for the illegal trade of live individuals and their parts. To address the need for tools that improve surveillance and monitoring of its remaining populations, we have developed a genome‐enabled single nucleotide polymorphism (SNP) panel targeting this species. From a dataset of 58 complete jaguar genomes, we identified and selected highly informative SNPs for geographic traceability, individual identification, kinship, and sexing. Our panel, named “Jag‐SNP”, comprises 459 SNPs selected from an initial pool of 13,373,949 markers based on the inter‐biome *F*_ST_, followed by rigorous filtering and addition of eight sex‐linked SNPs. We then randomly selected subsets of this panel and identified an 84‐SNP set that exhibited a similar resolving power. With both the 459‐SNP panel and its 84‐SNP subset, samples were assigned with 98% success to their biomes of origin and 65%–69% of them were assigned to within 500 km of their origin. Furthermore, *ca.* 10–18 SNPs within these panels were sufficient to distinguish individuals, whereas 6 sex‐linked SNPs perfectly separated males and females. We used whole‐genome data from an additional 18 jaguars to further test these panels, which provided insights into kinship relationships and allowed inference of geographic origin of samples collected outside the spatial scope of the original sample set. These results support the strong potential of these panels as an efficient tool for application in forensic, genetic, ecological, behavioral and conservation projects targeting jaguars.

## Graphical abstract

We have developed a genome‐enabled single nucleotide polymorphism (SNP) panel for use in jaguar geographic traceability, individual identification, kinship, and sexing. Our panel, named “Jag‐SNP”, comprises 459 SNPs selected from an initial pool of 13,373,949 markers based on the inter‐biome *F*_ST_, followed by rigorous filtering and addition of eight sex‐linked SNPs. We demonstrated that the panel and much smaller subsets of these markers can be successfully applied to all their target goals, supporting their effectiveness as multipurpose tools for forensic, genetic, ecological, behavioral and conservation projects targeting jaguars.

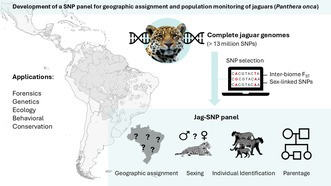

## RESUMO

O manejo da vida selvagem exige ferramentas poderosas para monitorar e proteger a biodiversidade à medida que esta diminui rapidamente. Nesse sentido, a área da genômica trouxe novas possibilidades para o desenvolvimento de ferramentas baseadas em polimorfismos de nucleotídeo único (SNPs), que melhoram as estimativas genéticas em relação a marcadores tradicionais, como os microssatélites. A aplicação de painéis de SNPs selecionados e otimizados a partir de grandes conjuntos de dados genômicos tem um grande potencial no contexto dos esforços de conservação de espécies esquivas e ameaçadas, como a onça‐pintada ( *Panthera onca* ), o maior felídeo das Américas e espécie guarda‐chuva para conservação da biodiversidade. Neste estudo, a partir de um conjunto de dados de 58 genomas completos de onças‐pintadas do Brasil, identificamos e selecionamos SNPs altamente informativos para rastreabilidade geográfica, identificação individual, parentesco e sexagem. Através de uma rigorosa filtragem, foram escolhidos 459 SNPs dentre 13.373.949 marcadores, com base no *F*_ST_ inter‐bioma, para compor um painel junto de outros 8 SNPs ligados ao sexo. Deste painel, selecionamos subconjuntos de forma aleatória e identificamos um com apenas 84 SNPs que apresentou um poder de resolução semelhante ao painel completo. Quanto à atribuição de origem geográfica, tanto o painel geral quanto o subconjunto atingiram uma acurácia de 98% relativa ao bioma de origem das amostras e 65–69% delas foram atribuídas a um raio de até 500 km da sua origem. Para identificação individual, observamos que 10–18 SNPs dentre os marcadores selecionados são suficientes para diferenciar indivíduos, enquanto 6 SNPs ligados ao sexo são capazes de separar corretamente machos e fêmeas. Por fim, através de uma validação in silico, também foi possível recuperar corretamente as relações de parentesco entre os indivíduos testados, ressaltando a versatilidade desses marcadores e seu potencial para o desenvolvimento de ensaios de genotipagem como ferramenta para perguntas forenses, genéticas e ecológicas envolvendo onças‐pintadas.

## 1 Introduction

Wildlife management demands powerful and reliable tools to monitor and protect biodiversity. Considering the complexity of studying endangered animals with naturally low densities and large home ranges, such as many species of large mammals, approaches must be able to use minimally invasive sampling methods (e.g., collection of hair, feces, feathers, saliva) and/or museum specimens. Moreover, samples confiscated from the wildlife trade may be processed, cooked, dried, or mixed with other species. In all such cases, these samples contain DNA with low quality and quantity, presenting considerable challenges to the generation of reliable genotype data for forensic or population genetic studies.

The field of genomics has brought new possibilities for the development of simple and cost‐effective tools (Hohenlohe et al. 2021; Natesh et al. 2019) that allow the use of samples containing lowamounts and low‐quality DNA (Andrews et al. 2018; Carroll et al. 2018; von Thaden et al. 2020), whereas also potentially improving the accuracy of genetic estimates relative to those obtained from traditional, low‐throughput genetic markers, such as microsatellites (a.k.a. STRs) (Glover et al. 2010; Morin et al. 2004; Weinman et al. 2015). Approaches based on single nucleotide polymorphisms (SNPs) present practical advantages since these markers are relatively easy to score and have low mutation rates (due to the bi‐allelic nature of selected sites) and low genotyping error. Their assays can be highly reproducible and easy to standardize across laboratories, so they can be readily incorporated into shared databases (Morin et al. 2004), making them an efficient method for applied conservation genomic efforts.

Although a larger set of SNP loci is required to achieve the same statistical power as microsatellites, the emergence of next‐generation sequencing platforms, such as SNP arrays (e.g., Fluidigm, Amplifluor, and MassARRAY), amplicon‐based target enrichment methods (e.g., GT‐seq) and in‐solution hybridization enrichment methods (e.g., SureSelect and myBaits) has contributed to overcoming this limitation. These methods require only small amounts of DNA (nanograms) and can assess many loci and samples simultaneously within a few hours (Campbell et al. 2015; Carroll et al. 2018; von Thaden et al. 2017). As a consequence, the use of SNP panels optimized from large genomic datasets is growing in wildlife research, for example, to assess population structure or hybridization, geographic provenance, individual identity, sex, and parentage (e.g., Buchalski et al. 2022; Ciezarek et al. 2022; Ekblom et al. 2021; Erwin et al. 2021; Jiang et al. 2020; Kleinman‐Ruiz et al. 2017; Kraus et al. 2015; Magliolo et al. 2021; Ogden and Linacre 2015; Stronen et al. 2022).

The application of such methods holds great potential in the context of conservation efforts directed to elusive and threatened species, such as the jaguar ( *Panthera onca* ). As the largest extant felid in the Americas and highly adaptable to different environments, this species plays an important ecological role as a top predator (Sanderson et al. 2002). Like other large carnivores, jaguars occur at low densities and have slow reproductive rates, which makes them sensitive to human disturbances (Paviolo et al. 2016; Romero‐Muñoz et al. 2019; Thompson et al. 2021). With the decrease in natural prey, frequent human–jaguar conflicts, and the increase of poacher activities in remote areas, all of which are driven by the increase of habitat loss and fragmentation, jaguars have already disappeared from almost 60% of their original range (Medellín et al. 2016; Quigley et al. 2018). Furthermore, recent evidence shows the existence of thriving domestic markets and the expansion of illegal trade of jaguar body parts among continents, including online sales of specimens, possibly as cheaper substitutes for the parts of increasingly scarce tigers and satisfying the demand for traditional Asian medicine (Arias 2021; Morcatty et al. 2020; Polisar et al. 2023).

There are several contextual and institutional factors that facilitate poaching and illegal trade of jaguars across its range, some of which include legal deficiencies, corruption, and a lack of financial, technological, and human resources for adequate enforcement (Arias 2021). In Brazil, which harbors the largest remaining populations of jaguars, the existence of clandestine illegal trophy hunting and organized criminal networks has been revealed, raising concerns about the need for tools to improve surveillance and monitoring of jaguar populations (Instituto Brasileiro do Meio Ambiente e dos Recursos Naturais Renováveis 2016; Operação Jaguar Da PF Desmantela Quadrilha de Caçadores Onça Pintada 2010).

To address these issues, in this study we used whole‐genome data from jaguars sampled across Brazilian biomes to select sets of SNPs that were capable of assigning samples to their probable source population, whereas also allowing individual‐level identification, sexing, and parentage assignment. We assessed the performance of these markers with several in silico approaches in the original set of individuals as well as by sequencing and extracting the respective genotypes from additional jaguar genomes. Our results indicate that the SNP panel developed in this study represents a promising tool to monitor and empower strategies against jaguar poaching, as well as to assess and manage its remaining populations.

## 2 Materials and Methods

### 2.1 Panel Design Using Whole‐Genome Data

**FIGURE 1:**
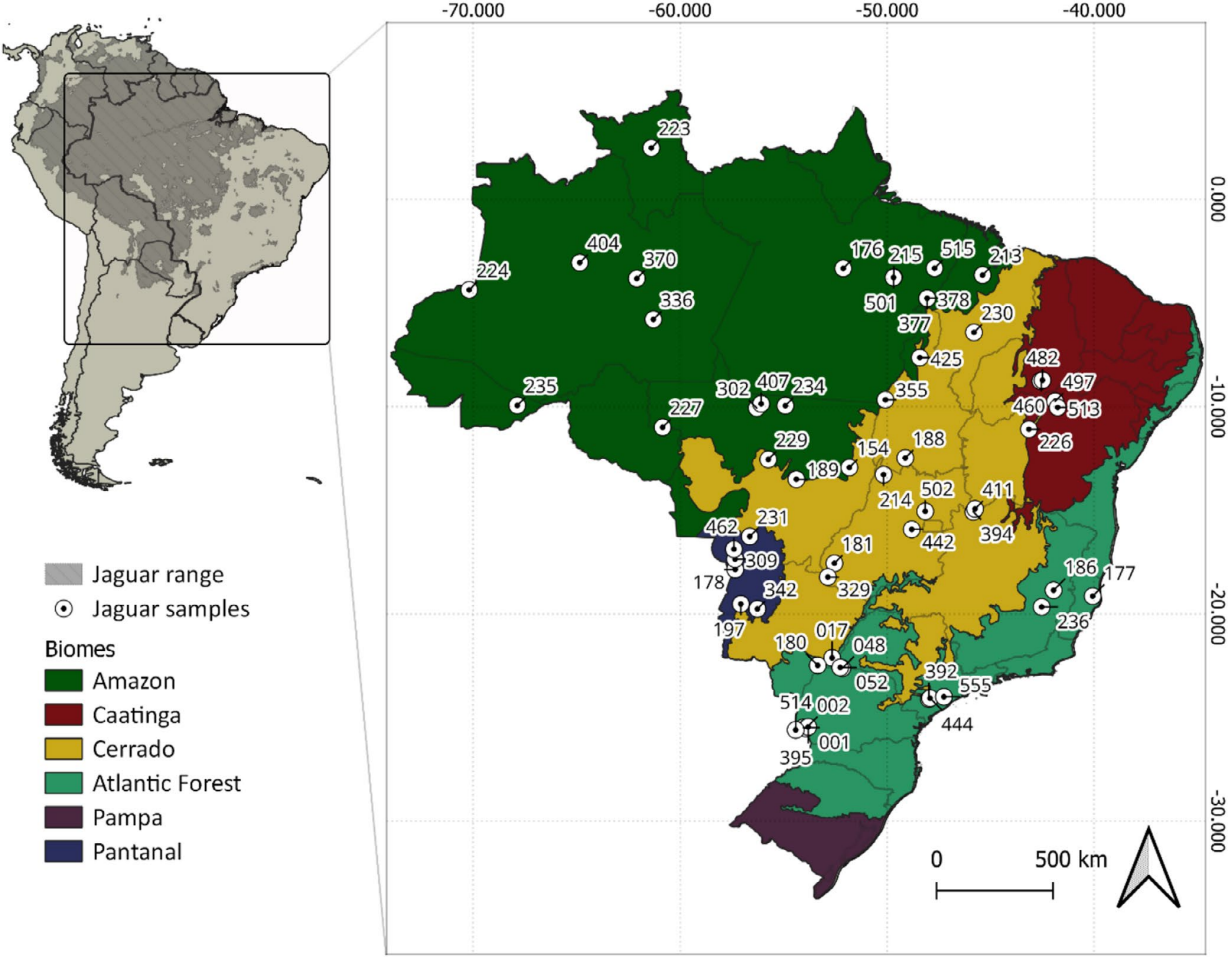
Current jaguar range in South America (left) and sampling locations of jaguar genomes in the biomes where the species still occurs in Brazil (right).

Our dataset comprised 58 whole genomes sequenced using the Illumina HiSeq platform (Sartor et al., unpublished data), achieving an average coverage depth of 13×. These genomes were previously sequenced from blood samples collected from wild and captive animals of known origin, encompassing all five Brazilian biomes where jaguars are still found: Amazon (*n* = 18), Atlantic Forest (*n* = 14), Cerrado (*n* = 14), Caatinga (*n* = 6), and Pantanal (*n* = 6) (Figure 1 and Table S1).

We used the raw reads from these whole‐genome sequences, initially assessing their read quality using FastQC v0.12 (Andrews 2023). Sequencing reads were then aligned to a jaguar reference genome (GCF_028533385.1 https://www.dnazoo.org/assemblies/panthera_onca), using the BWA‐MEM algorithm (Li 2012), facilitated by the BAM_pipeline module within PALEOMIX v2.0 (Schubert 2021). This comprehensive pipeline performed several preprocessing steps: it trimmed adapter sequences, filtered out low‐quality reads (quality threshold: Phred Score = 20), removed PCR duplicates, realigned indels, and generated coverage statistics with average depth histograms.

We then used the Genome Analysis Toolkit (GATK) v4.2.6.1 (McKenna et al. 2010), which employs a genotype likelihood approach, to identify variants individually on each sample using the HaplotypeCaller program, followed by joint genotype calling among all samples using GenomicsDBImport and GenotypeGVCFs. From the 13,373,949 identified SNPs, we applied hard filters with GATK's VariantFiltration tool: QD (QualbyDepth): < 2.0; QUAL: < 30; SOR (StrandOddsRatio) > 3.0; FS (FisherStrandBias) > 60.0; MQ (RMSMappingQuality) > 40.0; MQRS (MappingQualityRankSumTest) < −12.5; and RPRS (ReadPosRankSumTest) < −8.0. We then employed VCFtools v0.1.17 (Danecek et al. 2011) and BCFtools (Danecek et al. 2021) to retain only biallelic sites that met parameters of mapping quality (minQ > 30) and minimum and maximum depth (‐‐minDP and ‐‐min‐meanDP ≥ 5; ‐‐maxDP and ‐‐max‐meanDP ≤ 100). We removed SNPs and samples with ≥ 5% and ≥ 20% missing data, respectively, and also indels and sites with minor allele frequency below 5% (MAF ≥ 0.05). To prioritize loci that segregate independently of each other, we filtered sites for linkage disequilibrium (LD) with PLINK v1.90b6.21 (Purcell et al. 2007). Considering a sliding window of 50 SNPs, we removed one of each pair of SNPs if their LD value (*r* ^2^) was greater than 0.1, and advanced 10 SNPs in each step (‐‐indep‐pairwise 50 10 0.1) (Figure S1). We also removed SNPs in repetitive regions, using the annotation of repeats and the *intersect* tool from BEDTools (Quinlan and Hall 2010), and removed SNPs deviating from Hardy–Weinberg Equilibrium, with significance set to *p* < 0.001. After these filtering steps (Table 1), we retained 83,527 independent and high‐confidence SNPs distributed across the 19 jaguar chromosomes and employed this “83k” dataset in further analyses.

**TABLE 1:** Number of SNPs retained after each sequential filtering step.

SNP filtering step	Filtered SNPs	Remaining SNPs
SNP calling		13,373,949
Hard filters	411,832	12,962,117
VCFtools filters	9,906,612	3,055,505
Biallelic sites		
MAF		
minDP/min‐meanDP		
maxDP/max‐meanDP		
Missing data		
Linkage disequilibrium	2,904,008	151,497
Repetitive regions	66,799	84,698
Hardy–Weinberg Equilibrium	1171	83,527

### 2.2 Inter‐Biome Differentiation Assessment

To determine the genetic differentiation among jaguars from different regions, we classified individuals by their biomes of origin and utilized VCFtools (Danecek et al. 2011) to compute pairwise *F*_ST_ values (Weir and Cockerham 1984) between the groups using the “83k” dataset. We chose a biome‐based approach since current jaguar conservation strategies employ large‐scale biomes or ecoregions as operational management units, an approach that incorporates the concept of conserving populations that present local adaptation to different habitats. We tested different combinations of individuals, assessing the impact on *F*_ST_ values of removing samples from transitional areas or with imprecise provenance information.

Additionally, we employed VCFtools to calculate the inbreeding coefficient for each individual, using the formula *F* = (*O* − *E*)/(*N* − *E*), where *O* represents the observed number of homozygotes; *E* = the expected number of homozygotes; and *N* denotes the total number of genotyped loci. Furthermore, to characterize genomic diversity, we calculated the total number of heterozygous sites and divided them by genome size (for each individual separately and for all individuals). To do this, we applied a custom python script (https://github.com/henriquevf/PopGen_scripts/blob/main/heterozygosity_estimation_v2.py), using ANGSD and realSFS (Korneliussen et al. 2014) to compute the site frequency spectrum (SFS) and to derive heterozygosity from the estimated allele frequencies across the genome in nonoverlapping sliding windows of 200 kb.

To assess the influence of geographical separation on genetic similarity among jaguars, we assessed patterns of isolation by distance in our dataset. This approach involved the creation of genetic distance matrices among individuals, using p‐distances and the tool VCF2Dis v1.47 (https://github.com/BGI‐shenzhen/VCF2Dis). Concurrently, we calculated geographic distance matrices based on Euclidean distances, utilizing the R package ecodist (Goslee and Urban 2007). The correlation between these genetic and geographic matrices was subsequently tested using a Mantel test with 10,000 permutations, which assesses if samples from nearby locations are more genetically similar than would be expected by chance (Diniz‐Filho et al. 2013).

### 2.3 Informative SNP Selection

To select the most informative sites for geographic assignment, we ranked the 83k SNPS based on their pairwise *F*_ST_ values (from highest to lowest) between the sampled biomes and kept the 50 loci with the highest *F*_ST_ from each pairwise comparison, yielding a 459‐SNP panel (see Section 3). Then, based on the performance of this marker set, random subsampled sets were assessed, prioritizing those with the strongest biome differentiation based on principal component analysis (PCA), which was performed using PLINK v1.90b6.21 (Purcell et al. 2007) and plotted with ggplot2 in R v4.3.0 (Wickham 2016). Only the first two principal components were analyzed.

To select SNPs that were informative for sexing, we identified 698 SNPs located on the X chromosome and searched for sites that were close to the X‐linked genes *Amelogenin* (*AMELX*) and *Zinc‐finger protein* (*ZFX*), which have been used for sex identification of jaguars and other felids (Pilgrim et al. 2005). Subsequently, we analyzed the genotypes of these SNPs in each individual and visualized their distribution in males and females using a PCA.

### 2.4 Assessment of SNP Panel Performance

To perform population assignment tests, we employed a discriminant analysis of principal components (DAPC) with the ADEGENET v2.1.10 R package in R. This multivariate approach identifies principal components that optimally summarize the differences among clusters, thereby minimizing within‐cluster variation (Jombart et al. 2010). Initially, we determined the number of clusters using the *find.clusters* function, based on the lowest Bayesian information criterion (BIC) value. Subsequently, the optimal number of principal components (PCs) was ascertained using the *optim.a.score* command in conjunction with the cross‐validation function *xvalDapc*, considering that the number of PCs can significantly influence the outcomes of the analysis. Finally, the discriminant analysis (DA) was run using the *dapc* function. To account for unequal sample sizes of populations, we also employed the self‐assignment function in the package *rubias* (Moran and Anderson 2019), considering a robust assignment to be a > 0.8 posterior probability of assignment to the inferred genetic group.

In addition, to characterize population structure with our panels, we determined the global ancestry proportions for each individual. This was achieved using the model‐based approach that applies a maximum likelihood method implemented in Admixture v1.3 (Alexander et al. 2009). We conducted the analysis using the same number of *K* identified with DAPC.

We also conducted a continuous geographic assignment analysis with a Bayesian approach, utilizing the Markov Chain Monte Carlo (MCMC) method implemented in the program SCAT v3.0.2 (Smoothed and Continuous Assignment Tests) (Wasser et al. 2004). This program leverages allele frequencies and geographic information from reference samples, along with spatial smoothing techniques, to assign each unidentified individual to a location within the entire sampling area, using leave‐one‐out cross‐validation. We executed SCAT with a grid file covering the jaguar range in Brazil, conducting 100 sampled iterations (Niter) separated by 100 MCMC steps (Nthin), and following initial 100 burn‐in iterations (Nburn), which were subsequently discarded. To assess accuracy, we computed the Haversine distance between the assigned and actual locations using R v4.3.0.

We used CERVUS v3.0.7 (Kalinowski et al. 2007) to calculate the polymorphic information content (PIC) and the combined non‐exclusion probabilities for each locus selected for geographic assignment. PIC measures a marker's ability to detect polymorphism within a group of individuals, whereas non‐exclusion probabilities assess the average probability that the set of loci will not exclude one (NE‐1P) or a pair (NE‐PP) of unrelated candidate parents from the parentage of an arbitrary offspring, or the probability that the set will fail to distinguish between two randomly selected individuals (NE‐I) or full siblings (NE‐SI). To assess its performance for individual identification, we estimated and visualized probabilities of identity (PID and PID_sibs_) using GenAlEx v6.5 (Peakall and Smouse 2012) and the R package POPGENUTILS v0.1.8 (Tourvas et al. 2023). These probabilities represent the likelihood that two randomly chosen individuals from a population will have the same genotype across the assessed loci.

### 2.5 Validation With Additional Genomic Data

To further assess the power of our SNP panel, we sequenced 18 additional complete jaguar genomes, including seven captive‐bred and 11 wild‐born animals from Argentina, Brazil, and Paraguay. This dataset comprises 13 individuals with known pedigree relationships (8 parent–offspring, 4 sibling relationships) and 6 unrelated samples (Figure S2, Table S2), allowing us to test the power of the panel not only for geographic assignment (including animals sampled outside the scope of the original reference set of 58 samples), individual identification, and sexing, but also for parentage assignment and sibship reconstruction. These additional samples were preserved with TES buffer (100 mM Tris, 100 mM EDTA, 2% SDS) and stored at −20°C up to DNA extraction. We extracted genomic DNA using a modified phenol‐chloroform protocol (Sambrook et al. 1989), and the integrity and concentration of the extracts were verified on a 1% agarose gel stained with GelRed (Biotium). Whole‐genome fragment libraries were constructed with the TruSeq PCR‐free kit (Illumina), and sequencing was performed on Illumina NovaSeq S4 and NovaSeqX instruments. Read processing, mapping, and SNP calling for these new genomes were conducted as described above, and genotypes at the respective sites of our selected panels were extracted for analysis.

We used these novel genotypes to test the performance of our SNP panels, employing the same approaches described above with an independent set of individuals. We carried out an identity analysis with CERVUS, using 90% of loci as a minimum threshold required for a match and allowing only 5% mismatches. For geographic assignment, we only used samples of individuals born in the wild for which we had at least approximate provenance information (Table S2).

In addition, we used the new individuals with known genealogical relationships to test the performance of the panels in analyses of parentage and kinship. Based on the observed allele frequencies, we used CERVUS v3.0.7 to perform a simulation to determine critical values for logarithm of the odds (LOD) scores, that is, threshold values for determining whether a potential parent has a high enough likelihood to assign parentage. As a simulation, we used 100,000 offspring, an estimated genotyping error rate of 1%, a minimum number of loci typed as half of the total number of loci, and an estimated proportion of parents sampled of 20% (for each sex), to allow for the possibility of unsampled parents. We accepted only assignments with a 95% confidence level. For full‐sibling and half‐sibling assignments, maximum likelihood estimates of relatedness between the pairs of samples were conducted with ML‐Relate (Kalinowski et al. 2006), which also uses simulations to determine which relationships are consistent with genotype data, allowing pairwise hypothesis testing. In this case, we performed 1000 simulations for each pairwise test.

## 3 Results

### 3.1 Basic Population Genetic Statistics

Observed individual genome‐wide heterozygosities ranged from 0.0004 to 0.001, whereas the mean heterozygosity for the whole sample set was approximately 0.0008, with a standard deviation from the mean of 0.0001 (Figure S3). As expected, higher heterozygosity values were associated with lower inbreeding coefficients (which ranged from −0.016 to 0.472), and vice versa.

From an initial dataset of 13,373,949 SNPs, we identified a total of 83,527 high‐quality and independent SNPs after careful filtering (referred to as the “83k SNP dataset”). We retained only SNPs present in 95% of the individuals, with MAF ≥ 0.05, outside repetitive regions, and we removed one individual (sample 302) with more than 20% of missing data from further analyses.

Using this 83k SNP dataset, we assessed the genetic differentiation among the samples which were grouped according to their biomes of origin (Table 2; below the diagonal). Overall differentiation among biomes was low but detectable. We found that individuals from the Caatinga and Pantanal exhibited the greatest genetic divergence (*F*_ST_: 0.052), which is plausible given their locations at opposite extremes of the sampled area. In contrast, those from the adjacent Amazon and Cerrado showed the lowest genetic divergence (*F*_ST_: 0.009), as expected due to their historically high connectivity. The other pairwise *F*_ST_ comparisons ranged from 0.0224 to 0.0409. When we excluded some individuals from transitional areas (e.g., 213, 226) or with imprecise provenance information (e.g., 230, 529), *F*_ST_ values varied only at the third decimal place (Tables S3–S7), so we decided to keep the full set of samples to develop the panel.

**TABLE 2:** *F*_ST_ values between individuals grouped by biomes, based on 83k SNPs (whole‐genome data; below diagonal) and 459 SNPs (459‐SNP panel; above diagonal).

*F*_ST_ (mean)	Amazon (*n* = 17)	Atlantic Forest (*n* = 14)	Pantanal (*n* = 6)	Cerrado (*n* = 14)	Caatinga (*n* = 6)
Amazon	0	0.22661	0.20966	0.10924	0.22069
Atlantic Forest	0.035485	0	0.2495	0.18897	0.26552
Pantanal	0.022361	0.029139	0	0.20963	0.32326
Cerrado	0.009527	0.026218	0.023079	0	0.17448
Caatinga	0.03477	0.040905	0.052651	0.024103	0

The Mantel tests revealed some isolation by distance among all individuals (*r*: 0.46; *p* value: 0.001), as well as within biomes (Table S8). When each biome was assessed separately, the strongest correlation was observed in the Caatinga (*r*: 0.68; *p* value: 0.006) and the lowest in the Amazon (*r*: 0.44; *p* value: 0.002). The Pantanal was an exception, showing statistically nonsignificant values in this assessment of isolation by distance.

### 3.2 Development and Assessment of the SNP Panel

**FIGURE 2:**
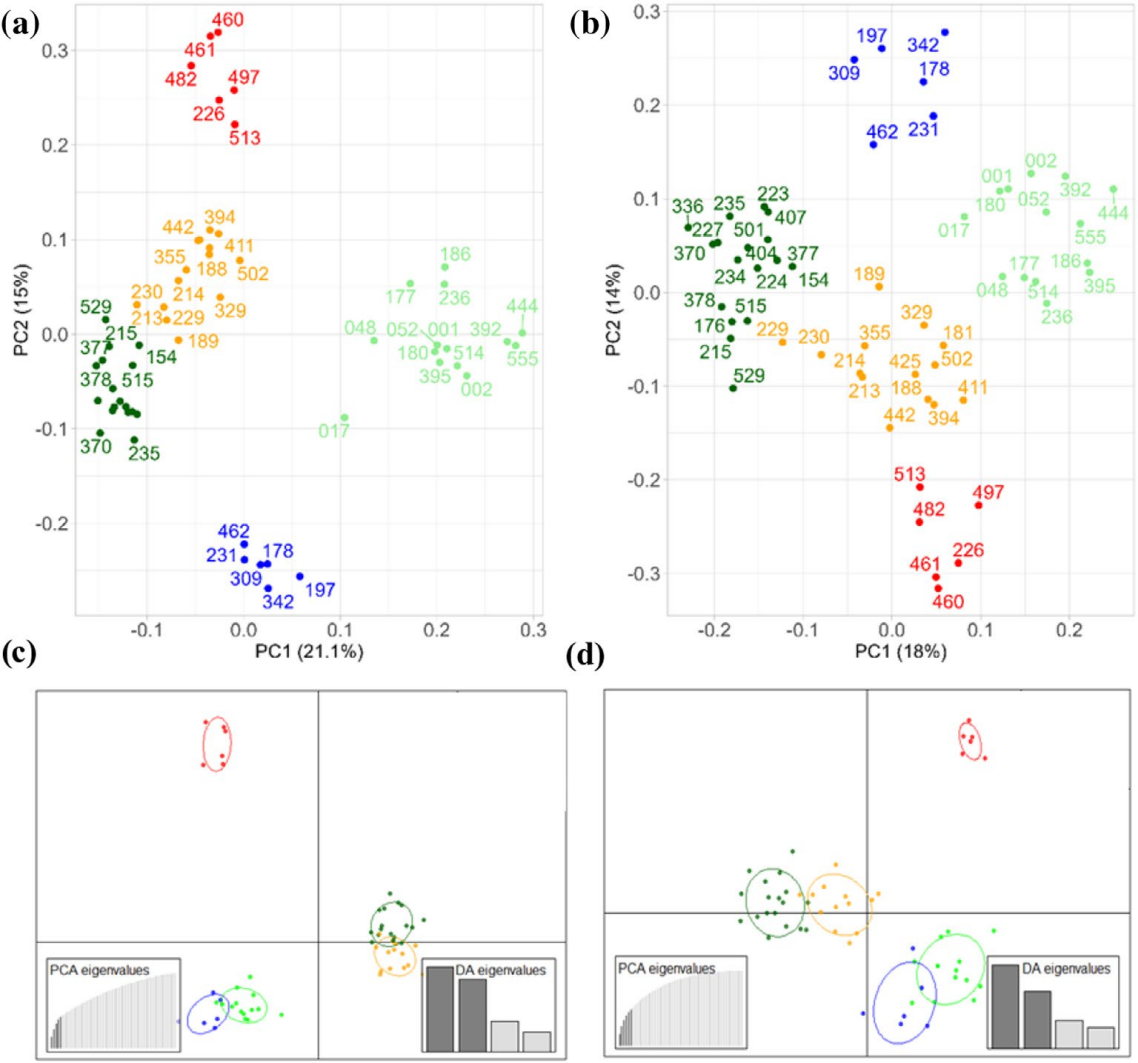
Principal component analysis (PCA) and discriminant analysis of principal components (DAPC) scatterplots of jaguar samples. (a) PCA calculated from the 459‐SNP panel, and (b) from a subset of this panel containing 84 SNPs. (c) DAPC scatterplots calculated from the 459‐SNP panel and (d) its derived subset. Only the first two axes of the DAPC are represented. Each point represents one sample, and the color denotes its biome of origin (Amazon—dark green; Atlantic Forest—light green; Caatinga—red; Cerrado—orange; Pantanal—blue).

From the 83k SNP dataset, we identified an *F*_ST_‐based panel comprising 459 SNPs (Figure 2a). This set included the top 50 SNPs of each pairwise biome comparison; 41 SNPs were retrieved in more than one comparison and were kept only once in this selected subset. We then randomly selected subsets of this 459‐SNP panel to assess whether smaller sets of SNPs (which can be genotyped more quicky and affordably) provide similar levels of information. As an example, we identified a set with only 84 SNPs that exhibited a similar resolving power (Figure 2b), correctly separating individuals by biomes of origin in a PCA.

Compared to the whole‐genome data (83k SNP panel), both panels identified similar patterns of genetic differentiation, but with much higher *F*_ST_ values. Individuals from the Caatinga and Pantanal exhibited the highest levels of divergence (*F*_ST_‐459: 0.32326 and *F*_ST_‐84: 0.34076), whereas individuals from the Amazon and Cerrado showed the lowest divergence (*F*_ST_‐459: 0.10924 and *F*_ST_‐84: 0.13407) (Tables 2 and 3). The other pairwise *F*_ST_ comparisons ranged from 0.17448 to 0.26552 for the 459‐SNP panel, and from 0.15541 to 0.25071 for the 84‐SNP panel. The mean expected heterozygosity was 0.4772 for the 459‐SNP panel (range: 0.149–0.5), and 0.487 for the 84‐SNP panel (range: 0.307–0.5). Our panels exhibited relatively high MAFs, with a mean of 0.427 (range: 0.08–0.5) for the 459‐SNP panel, and 0.440 (range: 0.187–0.5) for the 84‐SNP panel. The mean polymorphic information content (PIC) was 0.3592 for the 459‐SNP panel (range: 0.137–0.375), and 0.365 for the 84‐SNP panel (range: 0.258–0.375). Additionally, both panels showed low levels of combined non‐exclusion probabilities (Table 4).

**TABLE 3:** *F*_ST_ values between individuals grouped by biomes, based on 83k SNPs (whole‐genome data; below diagonal) and the 84‐SNP panel (above diagonal).

*F*_ST_ (mean)	Amazon (*n* = 17)	Atlantic Forest (*n* = 14)	Pantanal (*n* = 6)	Cerrado (*n* = 14)	Caatinga (*n* = 6)
Amazon	0	0.21362	0.2053	0.13407	0.25071
Atlantic Forest	0.035485	0	0.20795	0.15541	0.24138
Pantanal	0.022361	0.029139	0	0.21241	0.34076
Cerrado	0.009527	0.026218	0.023079	0	0.19701
Caatinga	0.03477	0.040905	0.052651	0.024103	0

**TABLE 4:** Summary statistics of the 459‐SNP and 84‐SNP panels.

Number of loci	459	84
Mean number of alleles per locus	2	2
Mean proportion of loci typed	0.9882	0.9877
Mean expected heterozygosity (*H*_e_)	0.4772	0.4874
Minor allele frequency (MAF)	0.427	0.440
Mean polymorphic information content (PIC)	0.3592	0.3657
Combined non‐exclusion probability (first parent) (NE‐1P)	7.433E‐0025	0.00002745
Combined non‐exclusion probability (second parent) (NE‐2P)	3.034E‐0040	0.00000004
Combined non‐exclusion probability (parent pair) (NE‐PP)	5.872E‐0064	1.718E‐0012
Combined non‐exclusion probability (identity) (NE‐I)	3.458E‐0187	1.577E‐0035
Combined non‐exclusion probability (sib identity) (NE‐SI)	6.042E‐0099	4.326E‐0019

Without predefined groups, we performed a DAPC with both panels, using *K* = 5 (the lowest BIC value) (Figure S5a,b). The k‐means clustering procedure correctly assigned all individuals to their expected biome of origin, except for individual 229 (which was sampled in a transitional area—see Figure 1). This individual was assigned to the Amazon rather than the Cerrado when using the 84‐SNP panel (Figure S4), consistent with findings from the PCA analysis. The cross‐validation process to determine the optimal number of PCs revealed a high success rate in outcome prediction when using 5–10 PCs (Figure S5e,f), associated with the lowest root mean squared error. Therefore, we retained the first 5 and 6 PCs for the 459‐SNP and the 84‐SNP panels, respectively, as recommended by the *optim.a.score* function and the four principal eigenvalues (Figure S5c,d). The discriminant analysis revealed clear differentiation across the five clusters (Figure 2c,d), with the *x* axis more distinctly separating individuals by biome using 84 SNPs compared to 459 SNPs, whereas the *y* axis distinguished among three groups: Pantanal + Atlantic Forest, Amazon + Cerrado, and Caatinga. Individuals from the Caatinga formed a distinctly isolated group, separated from the other clusters. The proportion of successful reassignment of individuals to their original clusters (based on the discriminant functions) is presented in Figure S4. The same results were found using the self‐assignment in *rubias*, with posterior probabilities > 0.8.

**FIGURE 3:**
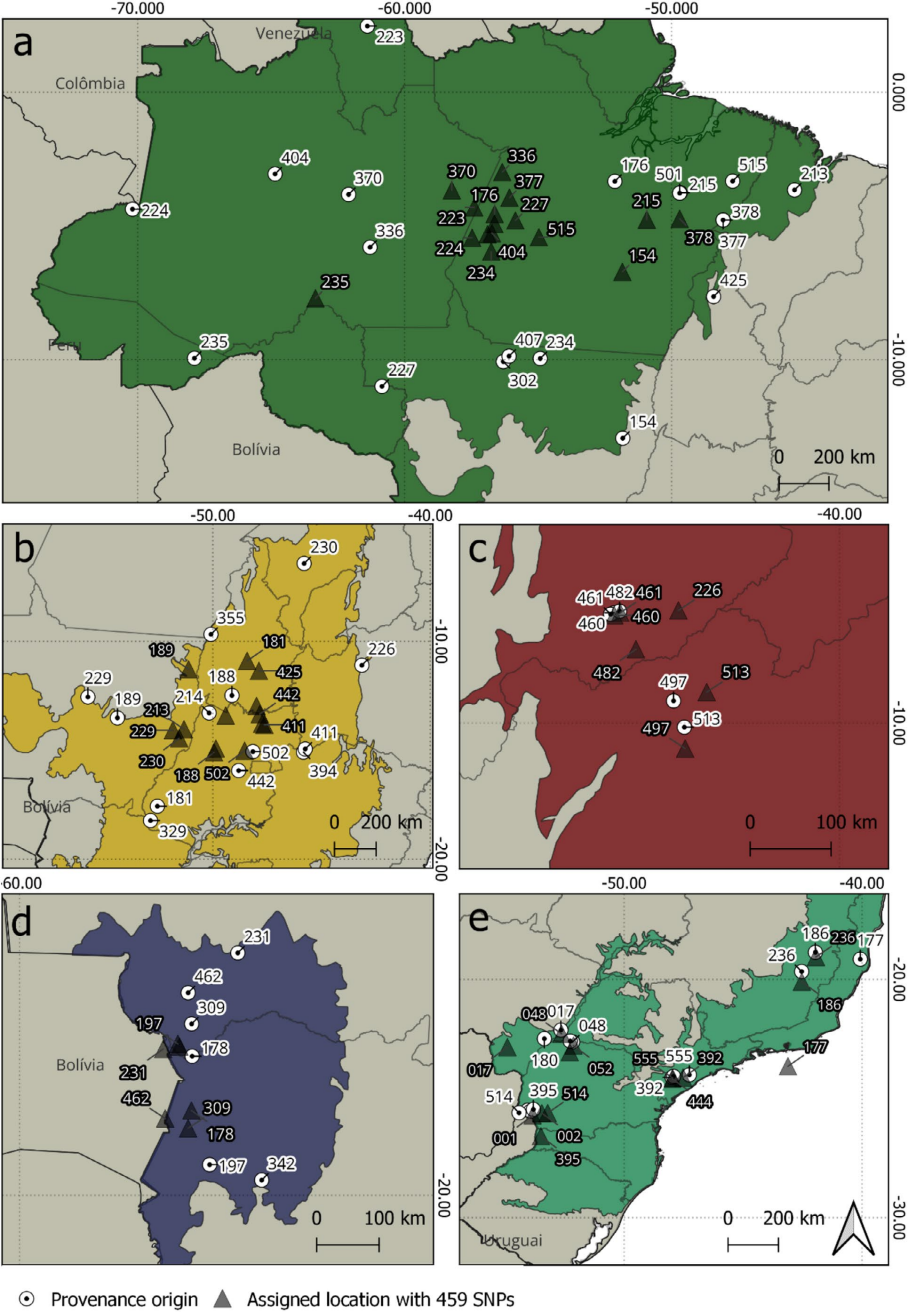
Geographic assignment with SCAT using the 459‐SNP panel. (a) Amazon; (b) Cerrado; (c) Caatinga; (d) Pantanal; and (e) Atlantic Forest. Circles indicate the actual origin of a sample, whereas triangles indicate its assigned location.

Following this analysis, we tested both panels for geographic assignment using the software SCAT, achieving an accuracy of 98% relative to the original biome of the samples (Figure 3 and Figure S6). There were very few cases of incorrect assignments. Specifically, using the 459‐SNP panel, individual 177 was incorrectly assigned to the ocean, located 591.44 km away from the known original coordinate in Atlantic Forest. This individual originates from the Reserva Natural Vale in Linhares, eastern Brazil, an isolated population characterized by strong differentiation due to high anthropogenic genetic drift (Srbek‐Araujo et al. 2018). Also, using the 84‐SNP panel, transitional individual 229 was assigned to the Amazon, 738.93 km away from its origin in the Cerrado.

The mean distance in kilometers between the actual and the assigned origins was larger for individuals from extensive biomes such as the Amazon, averaging 707.67 km with the 459‐SNP panel and 926.90 km with the 84‐SNP panel. This was followed by individuals from the Cerrado, with mean distances of 492.62 km for the 459‐SNP panel and 467.42 km for the 84‐SNP panel. For the Pantanal, the distances were 195.56 and 214.56 km for the 459‐SNP and 84‐SNP panels, respectively. For the Atlantic Forest, the mean distances were 125.36 and 164.89 km for the 459‐SNP and 84‐SNP panels, respectively. Finally, in the Caatinga, the distances averaged 80.45 km for the 459‐SNP panel and 114.45 km for the 84‐SNP panel. The most precise assignment occurred for individual 460, assigned only 3–18 km away from its original coordinates with our panels. Summary statistics by biome and for each individual assignment, as well as a comparison between assignments with DAPC, *rubias*, and SCAT, are presented in Tables S9–S11.

**FIGURE 4:**
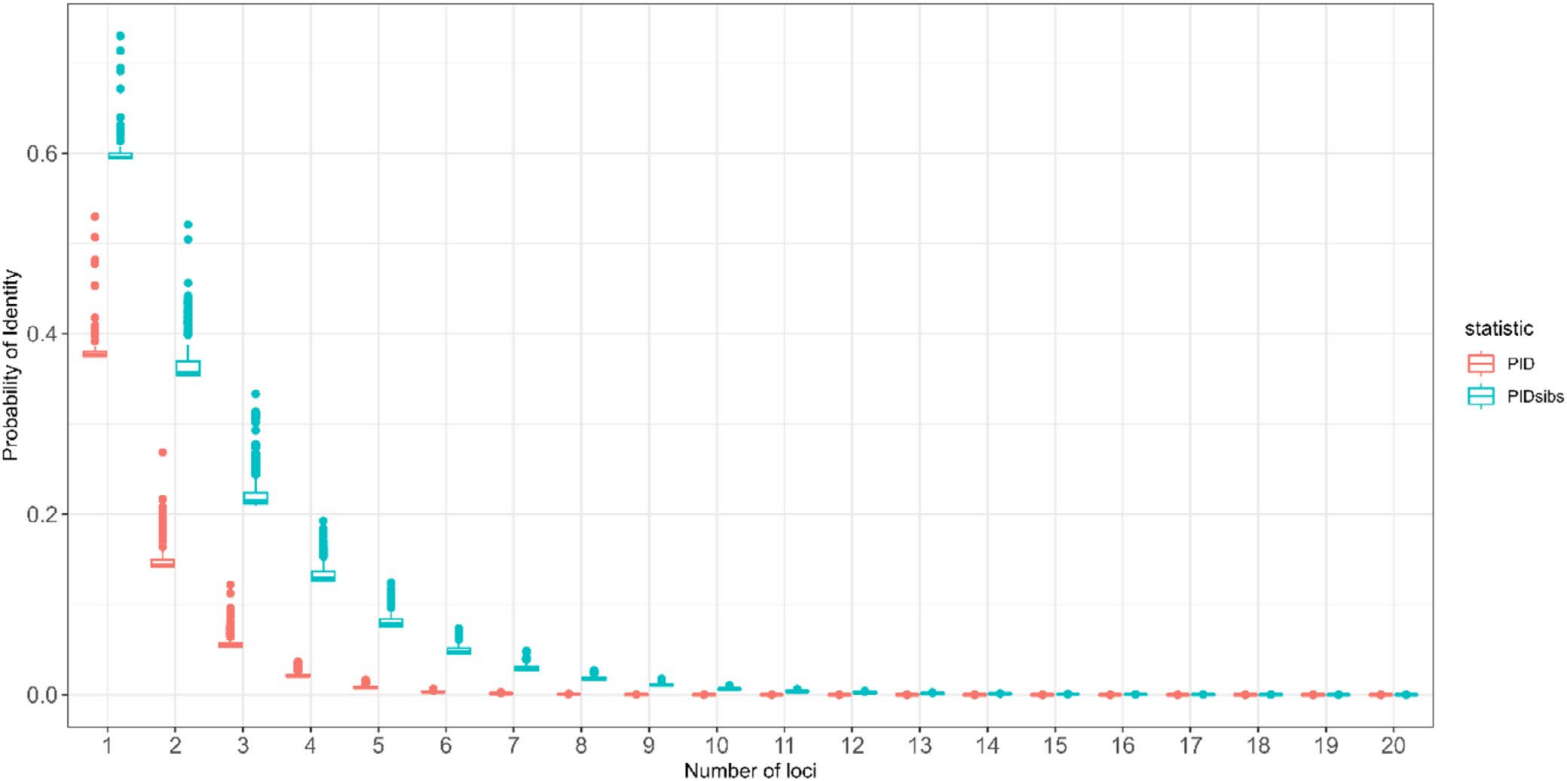
Probability of identity (PID) and probability of identity among siblings (PID_sisbs_) with an increasing number of loci within our 459‐SNP panel.

We explored the power of the selected markers for individual identification, employing a probability of identity (PI) cutoff of 0.0001 (Waits et al. 2001). We found that a panel of approximately 10 SNPs (PID: 7 × 10^−5^) provided adequate information to distinguish individuals, and approximately 18 SNPs (PID_sibs_: 1 × 10^−5^) were enough to differentiate related individuals (Figure 4). The PID per locus for the 459‐SNP and 84‐SNP panels varied from 0.375 to 0.737 (mean: 0.395) and from 0.375 to 0.529 (mean: 0.386), respectively. Meanwhile, the PID_sibs_ per locus varied from 0.594 to 0.860 (mean: 0.612) for the 459‐SNP panel and from 0.593 to 0.730 (mean: 0.604) for the 84‐SNP panel.

**FIGURE 5:**
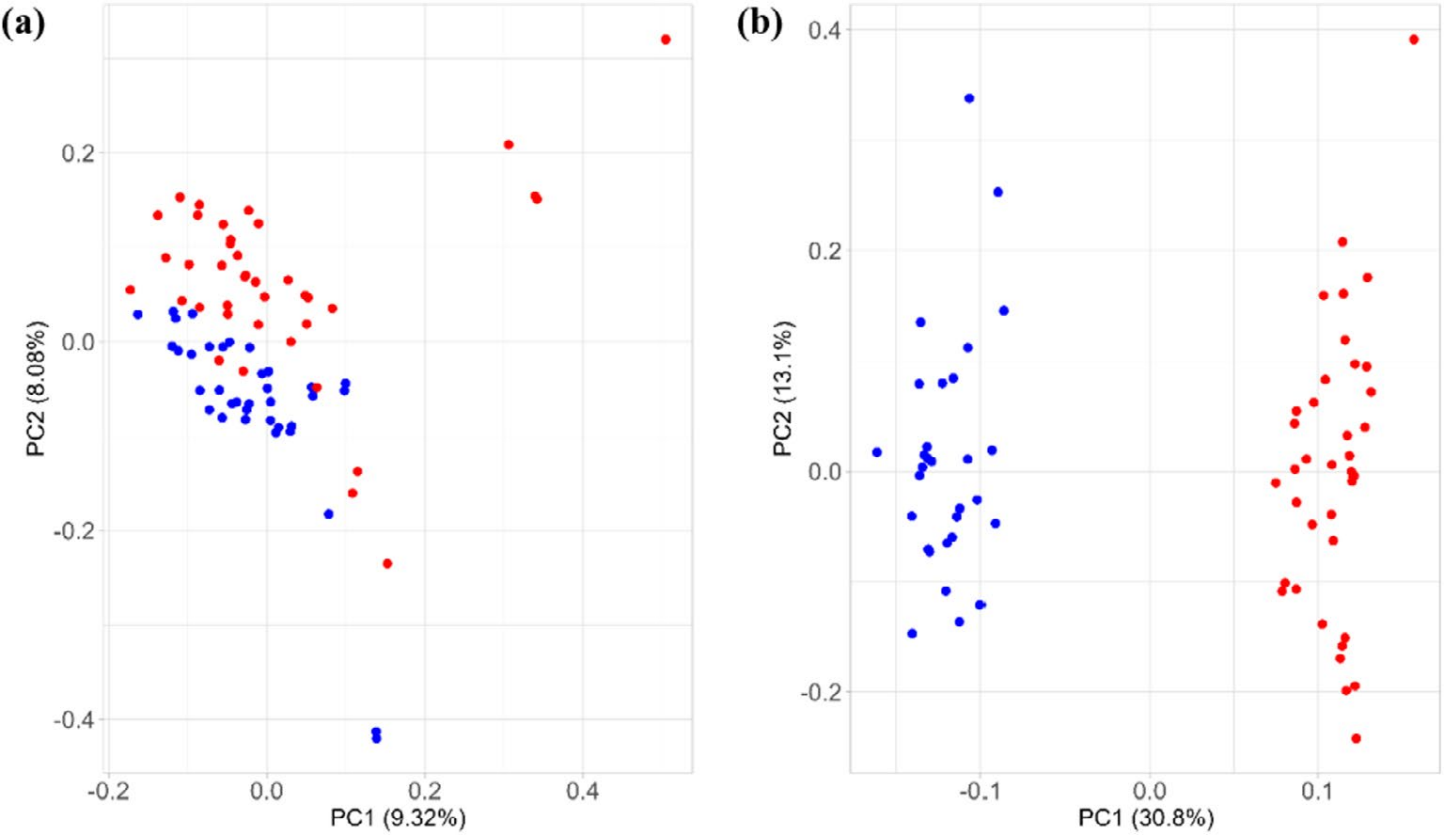
PCA performed with 698 X‐linked SNPs (a) and a subset of 20 SNPs (b), including all reference and validation jaguar samples. Individuals colored in red and blue are females and males, respectively.

For sexing, we explored the 698 SNPs identified on the X‐chromosome by assessing their genotypes, and found 20 SNPs that perfectly separated individuals by sex in a PCA, with PC1 explaining 30.8% of the variance (Figure 5). We selected 6 SNPs among these that showed a clear‐cut pattern in which all females were homozygous and males were either heterozygous or homozygous for the alternative allele. These SNPs were located in the X‐linked genes *ZFX*, *FRMPD4*, *TRAPPC2*, *TXLNG*, and *USP9X*.

### 3.3 Validation Using Additional Whole‐Genome Sequences

**FIGURE 6:**
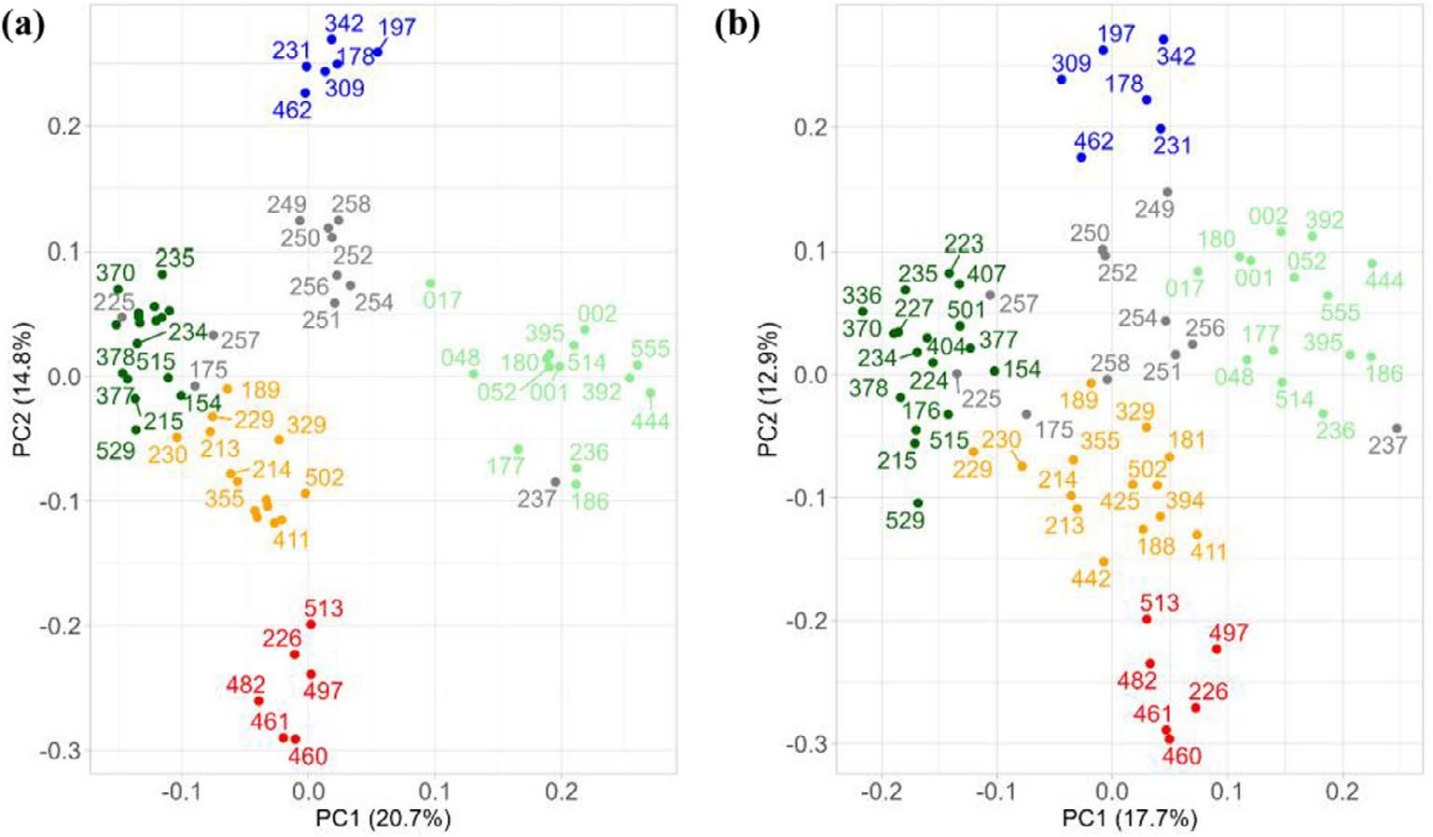
Principal component analysis (PCA) of jaguar samples, including 11 wild‐born individuals used for validation (gray), using (a) 459 SNPs and (b) 84 SNPs. Each point represents one sample, and the colors denote the biomes of origin (Amazon—dark green; Atlantic Forest—light green; Caatinga—red; Cerrado—orange; Pantanal—blue).

We used 18 additional whole‐genome sequences to further assess and validate our panels. With a minimum of 90% of loci required for a match and allowing only 5% of mismatches, the identity analysis revealed that both panels were capable of distinguishing all individuals from this additional dataset, even including related individuals. When comparing the genotypes of all individuals with each other, there were at least 155 mismatches (33%) and a maximum of 287 matched loci (62%) between individuals using the 459‐SNP panel, whereas there were at least 23 mismatches (27%) and a maximum of 61 matched loci (72%) using the 84‐SNP panel. With a PCA, we observed that, with the exception of individual 249 (from the Pantanal), three of the 11 wild‐born individuals from Brazil (or that had Brazilian parents) used for validation grouped with the Amazon cluster, whereas one sample grouped with the Atlantic Forest cluster, as expected (Figure 6). The other six individuals (which were sampled in Argentina and Paraguay) formed a distinct group, which was more clearly visualized using the 459‐SNP panel than with the 84‐SNP panel.

With regards to geographic assignment using both panels and a reference sample set of 56 individuals (from the original genome set), validation samples 175, 225, and 237 (all from Brazil) were assigned to their approximate regions of origin (*ca*. 5–105 km away), probably because the reference panel had individuals from very close locations, or even related ones (in the case of 175) (Table S12). The Pantanal individual 249 was better assigned with the 84‐SNP panel (340 km away from its origin) than with the 459‐SNP panel (580 km away) (Figures S7 and S8). Samples originating from Paraguay (250, 251, 252 and 258) were assigned to regions corresponding to Bolivia, Brazil, and Paraguay (*ca*. 230–750 km away from their respective origins), whereas samples 254 and 256, originating from El Impenetrable National Park (Argentina), were assigned to 121–848 km away from the known origin.

We then assessed genealogical relationships among individuals with a known history of ex situ breeding. In our analysis with CERVUS, when both parents were included, all mothers and fathers were correctly assigned at the 95% confidence level to the offspring using both panels. The mean LOD score for these assignments was 31.43 for the 459‐SNP panel and substantially higher, at 163.74, for the 84‐SNP panel. This analysis accurately assigned all four offspring to the correct parent pairs. However, in a case where only one parent's information was available (individual 260), the software was unable to precisely identify relationships, confusing its mother (261) and daughter (262). Additionally, unrelated samples were correctly not attributed to any parent, even with relaxed levels of confidence.

In our analysis with ML‐Relate, even when only one parent was included, it was possible to assign the offspring to their known parent with the 84‐SNP panel. Except for individuals 264 (not assigned to his mother) and 255 (her expected father was designated as her half sibling), all other relationships were correctly recovered with this panel. With respect to the siblings, with the 84‐SNP panel, the pairs of individuals 250–252 and 175–176 were designated as full siblings, whereas 255–264 and 262–263 were designated as half siblings. However, another eight pairs of individuals were incorrectly assigned as half‐siblings instead of unrelated (but the log‐likelihood of the individuals being unrelated were an average of only 1.17 less than the log‐likelihood of them being half‐siblings). In contrast, parentage analysis with the 459‐SNP panel assigned all expected parent‐offspring relationships as half‐siblings, with the exception of individual 263 and her father 254. Regarding the siblings, it was possible to assign them correctly, except for the half sibling pair 255–264, which was inferred to be unrelated.

## 4 Discussion

Despite the low genetic structure observed for jaguars (Eizirik et al. 2001; Kantek et al. 2021; Lorenzana et al. 2020; Sartor et al., unpublished data), our study showed that even a small set of highly informative SNPs is capable of successfully assigning samples to their biomes and approximate locations of origin, as well as providing sufficient information to identify individuals, sex, and kinship relationships. At the same time, since our SNP panels were designed from jaguars sampled only in Brazil, their utility for the whole species' range might be affected by ascertainment bias, thus requiring further verification (Albrechtsen et al. 2010). An initial step of this assessment was performed here, with validation samples collected in Argentina and Paraguay, and supported the panels' informative power even outside the original scope of the reference sample set.

Although some individuals (most of them from the Amazon) were assigned to 1000 km or more away from their sampling location, 65%–69% of samples were assigned to within 500 km of their actual origin with our SNP panels. Similarly to other studies, the highest accuracy occurred in the most isolated groups, which is the case for many individuals with low genetic diversity from highly fragmented areas, whereas samples from continuous habitats had less accurate assignments, due to high gene flow and correlated allele frequencies (Puckett and Eggert 2016; Wasser et al. 2015). In this context, we expect resolution to be further improved in larger areas, such as the Amazon and the Cerrado, with the collection of additional samples from locations not represented in our study. Meanwhile, it may be necessary to combine more than one statistical method to achieve the most reliable origin assignment.

It is also noteworthy that jaguar spatial ecology may be considered when interpreting differences between real and assigned locations. Jaguars are known to disperse over large distances (Morato et al. 2016), a process that might explain mismatches in geographic assignments. This could be especially true for males, which are more likely to disperse than females (Kantek et al. 2021). Moreover, jaguars present different patterns of space use and movement across regions with different degrees of habitat quality and human impacts. Jaguar mean home ranges have been documented for most Brazilian biomes (Atlantic Forest: 335.76 km^2^; Pantanal: 139.41 km^2^; Amazon: 286.2 km^2^; Cerrado: 1656.267 km^2^) and vary considerably among them, being larger in disturbed landscapes where the prey base tends to be reduced and risks related to human–wildlife conflict increase (Morato et al. 2016; Thompson et al. 2021). Thus, employing a buffer around the assigned location considering data on sex, home range, and habitat quality could lead to a more realistic assignment procedure.

Identification of individuals can be an important piece of information to quantify the number of animals entering illegal markets, as well as to survey and monitor wild populations through noninvasive methods. Interestingly, the number of SNPs necessary to perform individual identification estimated in this study was similar to those found for cheetahs, gray wolves, brown bears, and European wildcats (Magliolo et al. 2021; von Thaden et al. 2017, 2020), all of which were derived from a selection process based on the highest expected heterozygosity values (Means: jaguars = 0.48; cheetahs = 0.49; gray wolves = 0.29; brown bears: 0.31; European wildcat = 0.48). In contrast, our SNPs were selected based on their potential for geographic assignment (based on pairwise *F*_ST_ values), but they yielded a similar power to differentiate individuals. Furthermore, consistent with von Thaden et al. (2020), the same SNPs used for distinguishing individuals and assigning population origins also worked for reconstructing pedigree relationships, highlighting the multifunctionality of our panel.

Despite our panels having similarly high MAF values (459 SNPs: 0.427; 84 SNPs: 0.440), we observed that a larger number of markers did not necessarily improve the accuracy of kinship analyses with ML‐Relate and may even have hindered a more accurate assignment. It is possible that, with the employed analytical parameters, a larger amount of data implies excessive sensitivity due to the presence of some mismatches (beyond the expected threshold), leading the program to arrive at a more conservative conclusion of the assignment process. Additional tests, with varying parameters and different datasets, will be necessary to test this hypothesis. Furthermore, we also must take into account our small sample size per population, which can increase variance in allele frequency estimates and thus reduce the ability to distinguish close relatives. In conclusion, we provide here a jaguar SNP panel (Jag‐SNP) comprising 459 SNPs with efficient performance for geographic assignment, individual identification and parentage, along with 6 SNPs for sexing. We also demonstrate the effectiveness of a subset panel with only 84 SNPs, which illustrates the potential to subsample the 459‐SNP panel to optimize genotyping efficiency and reduce cost while still maintaining considerable informative power.

The practical implementation of these markers for jaguar genetics, forensics, and molecular ecology will require additional efforts to design and validate reliable genotyping assays targeting these loci, which should include assessments of sequencing efficiency across different sample types and genotyping platforms. If the aim of a study includes the geographical assignment of samples, we recommend designing an assay for the larger (459 SNP) panel, aiming to then select combinations of loci that can be pooled (multiplexed) together. On the other hand, if only the other questions (e.g., individual identification or kinship inference) are targeted, a subset such as the panel of 84 SNPs (plus sex‐linked markers) may be a cheaper option for assay design. Nevertheless, we strongly recommend that panels be standardized, aiming to promote the usage of the same markers across studies and in different countries, so that a common, public database can be constructed and consistently employed across the species' range. This is particularly important in the context of forensic analyses aiming to monitor and curb illegal trade, since such efforts will greatly benefit from a standardized database. It is also noteworthy that, given that forensic and non‐invasive samples are susceptible to allelic and locus dropout, designing short amplicons and implementing replicates and multiple runs of genotyping will be important components in the process of constructing reliable and replicable assays for these markers. Once these implementation steps are conducted and validated, we hope that the markers reported here will become a useful tool for jaguar forensic analysis and population monitoring, enabling improved management and conservation efforts on behalf of this iconic predator.

## Author Contributions

**Gabriele Zenato Lazzari:** conceptualization (equal), data curation (lead), formal analysis (lead), investigation (lead), methodology (equal), visualization (lead), writing – original draft (lead), writing – review and editing (equal). **Henrique Vieira Figueiró:** conceptualization (supporting), data curation (equal), formal analysis (supporting), validation (supporting), writing – review and editing (equal). **Caroline Charão Sartor:** conceptualization (supporting), data curation (equal), investigation (supporting), methodology (supporting), validation (supporting), writing – review and editing (equal). **Emiliano Donadio:** funding acquisition (equal), resources (equal), writing – review and editing (supporting). **Sebastián Di Martino:** funding acquisition (equal), resources (equal), writing – review and editing (supporting). **Hope M. Draheim:** conceptualization (supporting), investigation (supporting), validation (supporting), writing – review and editing (equal). **Eduardo Eizirik:** conceptualization (equal), funding acquisition (lead), project administration (lead), resources (lead), supervision (lead), validation (supporting), writing – original draft (equal), writing – review and editing (equal).

## Disclosure

Benefits generated: This study was led by scientists from the country providing most of the genetic samples (Brazil) and included collaborators from Argentina. All collaborators are included as co‐authors. The results of the research have been shared with sample providers, with wildlife authorities, and with the broader scientific community. The research addresses a priority concern, that is, improving conservation efforts on behalf of the focal species. More broadly, our group is committed to international scientific partnerships, environmental conservation, and institutional capacity building across the species' range and globally.

## Ethics Statement

All the samples analyzed in this study were obtained and processed under appropriate legal permits. Wild‐caught Brazilian jaguars were captured for field ecology studies under permits from the Ministério do Meio Ambiente (MMA), Instituto Brasileiro do Meio Ambiente e dos Recursos Naturais Renováveis (IBAMA) (Permit numbers: 12/2003, 003/2004, 19928‐1, 14202, SISBIO 30896, SISBIO 46031, SISBIO 47963, 70214‐2). Brazilian individuals kept in ex situ collections were sampled during routine checkups and do not require federal permits for such sampling (ICMBio/MMA, direct communication). Samples obtained in Argentina (including individuals from Paraguay) were imported to Brazil under CITES permit 22BR044634/DF.

## Conflicts of Interest

The authors declare no conflicts of interest.

## Supporting information


Data S1.

## Data Availability

SNP genotypes are deposited in Dryad (doi:10.5061/dryad.4tmpg4fkm); see reference Lazzari et al. [dataset] below. Raw sequence reads of novel genomes reported here are also deposited in the SRA (BioProject PRJNA348348).
