# Correction to “Development of a SNP Panel for Geographic Assignment and Population Monitoring of Jaguars (*Panthera onca*)”

**DOI:** 10.1002/ece3.71764

**Published:** 2025-07-07

Zenato Lazzari, G., Figueiró, H. V., Sartor, C. C., Donadio, E., Di Martino, S., Draheim, H. M., and Eizirik, E. 2025, “Development of a SNP Panel for Geographic Assignment and Population Monitoring of Jaguars (*Panthera onca*).” *Ecology and Evolution*, 15: e71465. https://doi.org/10.1002/ece3.71465.

The Acknowledgements section was incomplete. The following text should be included: “The findings and conclusions in this article are those of the author(s) and do not necessarily represent the views of the US Fish and Wildlife Service.”

We apologize for this error.

