# Supplementary material for: Development of a SNP Panel for Geographic Assignment and Population Monitoring of Jaguars (Panthera onca)
Source: Ecol Evol. 2025 May 22;15(5):e71465. doi: 10.1002/ece3.71465 (PMC12098306; doi:10.1002/ece3.71465)
Supplement: Supplementary file 1 — Data S1. [file ECE3-15-e71465-s001.docx]

Revised draft

Supporting information for online publication

**Development of a SNP panel for geographic assignment and population monitoring of jaguars (*Panthera onca*)**

Gabriele Zenato Lazzari¹, Henrique Vieira Figueiró¹^,^², Caroline Charão Sartor¹^,^³, Emiliano Donadio^4^, Sebastián Di Martino^4^, Hope M. Draheim^5^, Eduardo Eizirik^1,6^

Table S1 - Samples used to generate the initial reference panel employed in this present study. M – male; F – female; PN – National Park; PE – State Park; RDS - Sustainable Development Reserve

| Sample | Sex | Locality | State | Biome | Coord. precision | Latitude | Longitude |
| --- | --- | --- | --- | --- | --- | --- | --- |
| 001  002  017  048  052  154  176  177  178  180  181  186  188  189  197  213  214  215  223  224  226  227  229  230  231  234  235  236  302  309  329  336F  342  355  370  377  378  392  394  395  404  407  411  425  442  444  460  461  462  482  497  501  502  513  514  515  529  LegadoSP | M | PN do Iguaçu | PR | Atlantic Forest | Park | -25.494051 | -54.053932 |
| 002 | M | PN do Iguaçu | PR | Atlantic Forest | Park | -25.531432 | -53.929674 |
| 017 | M | Porto Primavera | MS | Atlantic Forest | Park | -22.1297222 | -52.65944444 |
| 048 | M | PE Morro do Diabo | SP | Atlantic Forest | Park | -22.6277778 | -52.16777778 |
| 052 | F | PE Morro do Diabo | SP | Atlantic Forest | Park | -22.5936111 | -52.26638889 |
| 154 | F | B. Rio das Mortes | MT | Amazon | Exact | -12.9409 | -51.8254 |
| 176 | F | Altamira | PA | Amazon | County | -3.33478 | -52.1233 |
| 177 | M | Reserva Natural Vale | ES | Atlantic Forest | Park | -19.1515 | -40.0708 |
| 178 | F | Corumbá | MS | Pantanal | Unknown | -17.8617 | -57.3411 |
| 180 | M | Taquarussu | MS | Atlantic Forest | Park | -22.49 | -53.356 |
| 181 | F | Mineiro | GO | Cerrado | County | -17.5665 | -52.5496 |
| 186 | M | Gov. Valadares | MG | Atlantic Forest | County | -18.8649 | -41.9599 |
| 188 | M | Alvorada | TO | Cerrado | County | -12.4829 | -49.1232 |
| 189 | F | Paranatinga | MT | Cerrado/Amazon | State | -13.5135 | -54.3866 |
| 197 | F | Passo do Lontra | MS | Pantanal | Exact | -19.5303 | -57.0719 |
| 213 | F | Santa Inês | MA | Cerrado/Amazon | County | -3.656358 | -45.390174 |
| 214 | M | S. Mig. Araguaia | GO | Cerrado | County | -13.286063 | -50.172959 |
| 215 | F | Tucuruí | PA | Amazon | County | -3.75906 | -49.6735 |
| 223 | F | Mucajaí | RR | Amazon | State | 2.476513 | -61.3948 |
| 224 | F | Atalaia do Norte | AM | Amazon | County | -4.373049 | -70.195183 |
| 226 | F | Barra | BA | Caatinga/Cerrado | County | -11.0963 | -43.1517 |
| 227 | F | Espigão D'Oeste | RO | Amazon | County | -11.0022 | -60.8477 |
| 229 | M | Sorriso | MT | Cerrado/Amazon | County | -12.5526 | -55.7439 |
| 230 | M | Fernando Falcão | MA | Cerrado | State | -6.42517 | -45.8051 |
| 231 | F | Poconé | MS | Pantanal | County | -16.2724 | -56.638 |
| 234 | F | Guarantã do Norte | MT | Amazon | County | -9.96146 | -54.9188 |
| 235 | F | Rio Branco | AC | Amazon | County | -9.950405 | -67.873767 |
| 236 | F | PE do Rio Doce | MG | Atlantic Forest | Exact | -19.671 | -42.5354 |
| 302 | M | Alta Floresta | MT | Amazon | County | -10.0765 | -56.3015 |
| 309 | M |  | MT | Pantanal | State | -17.364329 | -57.34928 |
| 329 | F | PN das Emas | GO | Cerrado | Park | -18.225511 | -52.866094 |
| 336 | M | Manicoré | AM | Amazon | County | -5.79768 | -61.2945 |
| 342 | F | Miranda | MS | Pantanal | Farm | -19.7666667 | -56.27 |
| 355 | F | PE do Cantão | TO | Cerrado | Exact | -9.677678 | -50.087271 |
| 370 | F | Codafós | AM | Amazon | County | -3.8243 | -62.0977 |
| 377 | M | Rondon do Pará | PA | Amazon | County | -4.7796 | -48.067 |
| 378 | F | Rondon do Pará | PA | Amazon | County | -4.7796 | -48.067 |
| 392 | F | PE Carlos Botelho | SP | Atlantic Forest | Park | -24.116441 | -47.982725 |
| 394 | F | PN Grande Sertão Veredas | MG | Cerrado | Exact | -15.0843 | -45.8375 |
| 395 | M | PN do Iguaçu | PR | Atlantic Forest | Park | -25.4605083 | -53.81778333 |
| 404 | F | RDS Mamirauá | AM | Amazon | Exact | -3.05802 | -64.85302 |
| 407 | F | Alta Floresta | MT | Amazon | County | -9.8725 | -56.087 |
| 411 | M | PN do Grande Sertão Veredas | BA | Cerrado | Exact | -14.9408 | -45.7548 |
| 425 | M | Nova Olinda | TO | Cerrado/Amazon | County | -7.642816 | -48.422234 |
| 442 | M | Corumbá de Goiás | GO | Cerrado | County | -15.931121 | -48.813073 |
| 444 | F | São Miguel Arcanjo | SP | Atlantic Forest | Exact | -24.0740833 | -47.92513889 |
| 460 | F | PN Serra da Capivara | PI | Caatinga | Exact | -8.75072 | -42.55626 |
| 461 | M | PN Serra da Capivara | PI | Caatinga | Exact | -8.766780556 | -42.59684444 |
| 462 | F | Estação Ecológica de Taiamã | MT | Pantanal | Exact | -16.88524 | -57.40286 |
| 482 | M | PN Serra da Capivara | PI | Caatinga | Exact | -8.73318333 | -42.49537778 |
| 497 | M | PN do Boqueirão da Onça | BA | Caatinga | Exact | -9.748516 | -41.883062 |
| 501 | M | Tucuruí | PA | Amazon | County | -3.77402 | -49.6928 |
| 502 | M | Mimoso de Goiás | GO | Cerrado | County | -15.0605 | -48.1595 |
| 513 | F | Sento Sé | BA | Caatinga | Exact | -10.0459 | -41.7607 |
| 514 | M | PN do Iguaçu | PR | Atlantic Forest | Exact | -25.60554 | -54.41986 |
| 515 | M | Paragominas | PA | Amazon | Exact | -3.32666667 | -47.70888889 |
| 529 | F |  |  | Amazon | Unknown |  |  |
| 555 | M | Legado das Águas | SP | Atlantic Forest | County | -24.008748 | -47.258942 |

Table S2 – Sample information for the 18 additional jaguar genomes used for panel validation analyses. M – male; F – female; PE – State Park;

| Sample | Sex | Approximate origin | Analyses | | | |
| --- | --- | --- | --- | --- | --- | --- |
|  |  |  | Parentage | Geographic assignment | Individual identification | Sexing |
| 175 | F | Xingu zone, Amazon, Brazil | X | X | X | X |
| 225 | M | Amazon, Brazil | X | X | X | X |
| 237 | M | PE Rio Doce, Brazil | X | X | X | X |
| 249 | M | Escola Jatobazinho, Corumbá, Brazil | X | X | X | X |
| 250 | M | Fazenda Faro Moro, Paraguay | X | X | X | X |
| 251 | F | Fazenda Faro Moro, Paraguay | X | X | X | X |
| 252 | M | Fazenda Faro Moro, Paraguay | X | X | X | X |
| 254 | M | El Impenetrable National Park, Argentina | X | X | X | X |
| 255 | F | Captive-born | X |  | X | X |
| 256 | M | El Impenetrable National Park, Argentina | X | X | X | X |
| 257 | F | Captive-born (Amazonian grandparents) | X | X | X | X |
| 258 | M | El, Olimpo, Paraguay | X | X | X | X |
| 259 | M | Captive-born | X |  | X | X |
| 260 | F | Captive-born | X |  | X | X |
| 261 | F | Captive-born | X |  | X | X |
| 262 | F | Captive-born | X |  | X | X |
| 263 | F | Captive-born | X |  | X | X |
| 264 | M | Iberá National Park, Argentina | X |  | X | X |

Table S3 - F_ST_ values between individuals grouped by biomes, based on 83k SNPs (whole-genome data), including individual 529 (below the diagonal) and removing this individual (above the diagonal).

| F_ST_ (mean) | Amazon | Atlantic Forest | Pantanal | Cerrado | Caatinga |
| --- | --- | --- | --- | --- | --- |
| Amazon | 0 | 0.035789 | 0.022306 | 0.010096 | 0.035787 |
| Atlantic Forest | 0.035485 | 0 | 0.029139 | 0.026218 | 0.040905 |
| Pantanal | 0.022361 | 0.029139 | 0 | 0.023079 | 0.052651 |
| Cerrado | 0.009527 | 0.026218 | 0.023079 | 0 | 0.024103 |
| Caatinga | 0.03477 | 0.040905 | 0.052651 | 0.024103 | 0 |

Table S4: F_ST_ values between individuals grouped by biomes, based on 83k SNPs (whole-genome data), including individual 213 (below diagonal) and removing this individual (above diagonal).

| F_ST_ (mean) | Amazon | Atlantic Forest | Pantanal | Cerrado | Caatinga |
| --- | --- | --- | --- | --- | --- |
| Amazon | 0 | 0.035485 | 0.022361 | 0.01104 | 0.03477 |
| Atlantic Forest | 0.035485 | 0 | 0.029139 | 0.026178 | 0.040905 |
| Pantanal | 0.022361 | 0.029139 | 0 | 0.023837 | 0.052651 |
| Cerrado | 0.009527 | 0.026218 | 0.023079 | 0 | 0.024423 |
| Caatinga | 0.03477 | 0.040905 | 0.052651 | 0.024103 | 0 |

Table S5 - F_ST_ values between individuals grouped by biomes, based on 83k SNPs (whole-genome data), including individual 226 (below diagonal) and removing this individual (above diagonal).

| F_ST_ (mean) | Amazon | Atlantic Forest | Pantanal | Cerrado | Caatinga |
| --- | --- | --- | --- | --- | --- |
| Amazon | 0 | 0.035485 | 0.022361 | 0.0095266 | 0.037331 |
| Atlantic Forest | 0.035485 | 0 | 0.029139 | 0.026218 | 0.043894 |
| Pantanal | 0.022361 | 0.029139 | 0 | 0.023079 | 0.057377 |
| Cerrado | 0.009527 | 0.026218 | 0.023079 | 0 | 0.027397 |
| Caatinga | 0.03477 | 0.040905 | 0.052651 | 0.024103 | 0 |

Table S6 - F_ST_ values between individuals grouped by biomes, based on 83k SNPs (whole-genome data), including individual 230 (below diagonal) and removing this individual (above diagonal).

| F_ST_ (mean) | Amazon | Atlantic Forest | Pantanal | Cerrado | Caatinga |
| --- | --- | --- | --- | --- | --- |
| Amazon | 0 | 0.035485 | 0.022361 | 0.010636 | 0.03477 |
| Atlantic Forest | 0.035485 | 0 | 0.029139 | 0.02608 | 0.040905 |
| Pantanal | 0.022361 | 0.029139 | 0 | 0.023337 | 0.052651 |
| Cerrado | 0.009527 | 0.026218 | 0.023079 | 0 | 0.02492 |
| Caatinga | 0.03477 | 0.040905 | 0.052651 | 0.024103 | 0 |

Table S7 - F_ST_ values between individuals grouped by biomes, based on 83k SNPs (whole-genome data), including individuals 213, 226, 230 e 529 (below diagonal) and removing these individuals (above diagonal).

| F_ST_ (mean) | Amazon | Atlantic Forest | Pantanal | Cerrado | Caatinga |
| --- | --- | --- | --- | --- | --- |
| Amazon | 0 | 0.035789 | 0.022306 | 0.012924 | 0.038356 |
| Atlantic Forest | 0.035485 | 0 | 0.029139 | 0.026147 | 0.043894 |
| Pantanal | 0.022361 | 0.029139 | 0 | 0.024349 | 0.057377 |
| Cerrado | 0.009527 | 0.026218 | 0.023079 | 0 | 0.02903 |
| Caatinga | 0.03477 | 0.040905 | 0.052651 | 0.024103 | 0 |

Table S8 – Results of Mantel tests conducted with the ecodist R package, using 10,000 permutations to assess significance. Columns are labeled as follows: mantelr: Mantel correlation coefficient (r); pval1: one-tailed p-value (null hypothesis: r <= 0); pval2: one-tailed p-value (null hypothesis: r >= 0); pval3: two-tailed p-value (null hypothesis: r = 0); llim: lower confidence limit; ulim: upper confidence limit.

| Mantel tests | mantelr | pval1 | pval2 | pval3 | llim.2.5% | ulim.97.5% |
| --- | --- | --- | --- | --- | --- | --- |
| All biomes | 0.465192 | 0.001 | 1 | 0.001 | 0.4368071 | 0.5405528 |
| Amazon | 0.4418099 | 0.002 | 0.999 | 0.002 | 0.3715653 | 0.6730381 |
| AtlanticForest | 0.4577537 | 0.001 | 1 | 0.001 | 0.3619347 | 0.6411821 |
| Pantanal | 0.4821786 | 0.134 | 0.876 | 0.145 | -0.2442022 | 0.9830188 |
| Cerrado | 0.4776534 | 0.001 | 1 | 0.001 | 0.3875861 | 0.5981506 |
| Caatinga | 0.6802842 | 0.006 | 0.996 | 0.006 | 0.655283 | 0.8721233 |

Table S9 - Summary statistics of geographic assignment, representing the distances between the assigned location and the original location grouped by biomes.

| Panel | Biome | Mean | Standard deviation | Minimum | Maximum |
| --- | --- | --- | --- | --- | --- |
| 459 SNPs | Amazon | 707.67 | 360.51 | 178.62 | 1416.39 |
|  | Caatinga | 80.45 | 110.82 | 3.14 | 301.37 |
|  | Cerrado | 492.62 | 307.84 | 41.25 | 1091.47 |
|  | Atlantic Forest | 125.36 | 147.71 | 12.48 | 591.44 |
|  | Pantanal | 195.56 | 57.49 | 92.99 | 264.82 |
| 84 SNPs | Amazon | 926.90 | 423.48 | 289.76 | 1814.94 |
|  | Caatinga | 114.45 | 92.14 | 18.15 | 265.32 |
|  | Cerrado | 467.42 | 242.07 | 197.87 | 854.91 |
|  | Atlantic Forest | 164.89 | 248.97 | 34.91 | 924.90 |
|  | Pantanal | 214.56 | 40.33 | 142.99 | 268.14 |

Table S10 *-* Distances (in km) between the original coordinates and the median coordinates assigned with the software SCAT.

| Sample | Distance from original coordinate (km) | |
| --- | --- | --- |
|  | **459 SNPs** | **84 SNPs** |
| 001 | 32.17 | 34.91 |
| 002 | 47.75 | 49.53 |
| 017 | 244.76 | 924.90 |
| 048 | 61.56 | 45.59 |
| 052 | 25.42 | 63.38 |
| 154 | 692.71 | 813.48 |
| 176 | 518.04 | 406.29 |
| 177 | 591.44 | 496.64 |
| 178 | 92.99 | 142.99 |
| 180 | 126.41 | 109.59 |
| 181 | 865.78 | 527.71 |
| 186 | 150.06 | 182.05 |
| 188 | 304.31 | 224.01 |
| 189 | 433.79 | 392.60 |
| 197 | 213.24 | 220.89 |
| 213 | 1326.33 | 1127.52 |
| 214 | 83.71 | 197.87 |
| 215 | 178.62 | 935.32 |
| 223 | 876.67 | 1814.94 |
| 224 | 1416.39 | 1242.06 |
| 226 | 301.37 | 265.32 |
| 227 | 883.43 | 507.78 |
| 229 | 455.78 | 738.93 |
| 230 | 1091.47 | 307.84 |
| 231 | 205.06 | 268.14 |
| 234 | 489.58 | 915.47 |
| 235 | 557.80 | 693.24 |
| 236 | 92.95 | 65.12 |
| 309 | 178.74 | 219.26 |
| 329 | 785.24 | 699.44 |
| 336 | 632.41 | 455.23 |
| 342 | 264.82 | 212.95 |
| 355 | 583.80 | 505.76 |
| 370 | 429.94 | 289.76 |
| 377 | 893.38 | 1029.86 |
| 378 | 180.56 | 1291.96 |
| 392 | 46.82 | 35.79 |
| 394 | 251.28 | 220.80 |
| 395 | 129.51 | 88.11 |
| 404 | 935.21 | 1325.34 |
| 407 | 515.49 | 660.63 |
| 411 | 236.81 | 207.91 |
| 425 | 417.17 | 854.91 |
| 442 | 307.32 | 298.10 |
| 444 | 12.48 | 44.50 |
| 460 | 3.14 | 18.15 |
| 461 | 12.15 | 33.31 |
| 462 | 218.49 | 223.16 |
| 482 | 52.09 | 76.02 |
| 497 | 61.71 | 136.00 |
| 501 | 781.15 | 1208.15 |
| 502 | 41.25 | 293.87 |
| 513 | 52.22 | 157.93 |
| 514 | 120.89 | 82.50 |
| 515 | 838.96 | 728.04 |
| 555 | 72.77 | 85.85 |

Table S11 – Comparison between geographic assignments estimated with SCAT, DAPC (Discriminant Analysis of Principal Components) and the *rubias* R package. AF – Atlantic Forest; AM – Amazon; CE – Cerrado; CA – Caatinga; PA – Pantanal.

| Sample | Biome of origin | Assigned biome | | | | | |
| --- | --- | --- | --- | --- | --- | --- | --- |
|  |  | **SCAT** | | **DAPC** | | **rubias** | |
|  |  | **459 SNPs** | **84 SNPs** | **459 SNPs** | **84 SNPs** | **459 SNPs** | **84 SNPs** |
| 001 | AF | AF | AF | AF | AF | AF | AF |
| 002 | AF | AF | AF | AF | AF | AF | AF |
| 017 | AF | AF | AF | AF | AF | AF | AF |
| 048 | AF | AF | AF | AF | AF | AF | AF |
| 052 | AF | AF | AF | AF | AF | AF | AF |
| 154 | AM | AM | AM | AM | AM | AM | AM |
| 176 | AM | AM | AM | AM | AM | AM | AM |
| 177 | AF | Ocean/next to AF | AF | AF | AF | AF | AF |
| 178 | PA | PA | PA | PA | PA | PA | PA |
| 180 | AF | AF | AF | AF | AF | AF | AF |
| 181 | CE | CE | CE | CE | CE | CE | CE |
| 186 | AF | AF | AF | AF | AF | AF | AF |
| 188 | CE | CE | CE | CE | CE | CE | CE |
| 189 | CE/AM | CE | CE | CE | CE | CE | CE |
| 197 | PA | PA | PA | PA | PA | PA | PA |
| 213 | CE/AM | CE | CE | CE | CE | CE | CE |
| 214 | CE | CE | CE | CE | CE | CE | CE |
| 215 | AM | AM | AM | AM | AM | AM | AM |
| 223 | AM | AM | AM | AM | AM | AM | AM |
| 224 | AM | AM | AM | AM | AM | AM | AM |
| 226 | CE/CA | CA | CA | CA | CA | CA | CA |
| 227 | AM | AM | AM | AM | AM | AM | AM |
| 229 | CE/AM | CE | **AM** | CE | **AM** | CE | **AM** |
| 230 | CE | CE | CE | CE | CE | CE | CE |
| 231 | PA | PA | PA | PA | PA | PA | PA |
| 234 | AM | AM | AM | AM | AM | AM | AM |
| 235 | AM | AM | AM | AM | AM | AM | AM |
| 236 | AF | AF | AF | AF | AF | AF | AF |
| 309 | PA | PA | PA | PA | PA | PA | PA |
| 329 | CE | CE | CE | CE | CE | CE | CE |
| 336 | AM | AM | AM | AM | AM | AM | AM |
| 342 | PA | PA | PA | PA | PA | PA | PA |
| 355 | CE | CE | CE | CE | CE | CE | CE |
| 370 | AM | AM | AM | AM | AM | AM | AM |
| 377 | AM | AM | AM | AM | AM | AM | AM |
| 378 | AM | AM | AM | AM | AM | AM | AM |
| 392 | AF | AF | AF | AF | AF | AF | AF |
| 394 | CE | CE | CE | CE | CE | CE | CE |
| 395 | AF | AF | AF | AF | AF | AF | AF |
| 404 | AM | AM | AM | AM | AM | AM | AM |
| 407 | AM | AM | AM | AM | AM | AM | AM |
| 411 | CE | CE | CE | CE | CE | CE | CE |
| 425 | CE/AM | CE | CE | CE | CE | CE | CE |
| 442 | CE | CE | CE | CE | CE | CE | CE |
| 444 | AF | AF | AF | AF | AF | AF | AF |
| 460 | CA | CA | CA | CA | CA | CA | CA |
| 461 | CA | CA | CA | CA | CA | CA | CA |
| 462 | PA | PA | PA | PA | PA | PA | PA |
| 482 | CA | CA | CA | CA | CA | CA | CA |
| 497 | CA | CA | CA | CA | CA | CA | CA |
| 501 | AM | AM | AM | AM | AM | AM | AM |
| 502 | CE | CE | CE | CE | CE | CE | CE |
| 513 | CA | CA | CA | CA | CA | CA | CA |
| 514 | AF | AF | AF | AF | AF | AF | AF |
| 515 | AM | AM | AM | AM | AM | AM | AM |
| 555 | AF | AF | AF | AF | AF | AF | AF |

Table S12 – Validation samples used for geographic assignment using both SNP panels.

| Sample | Approximate origin | Latitude | Longitude | Distance from original coordinate (km) | |
| --- | --- | --- | --- | --- | --- |
|  |  |  |  | **Reference data (n =56)** | |
|  |  |  |  | **84-SNP panel** | **459-SNP panel** |
| 175* | Xingu zone, Amazon, Brazil (Near 176) | - | - | 105.83 | 17.82 |
| 225* | Amazon, Brazil (Near 224) | - | - | 16.01 | 5.51 |
| 237* | PE Rio Doce, Brazil (Near 236) | - | - | 66.53 | 43.21 |
| 249 | Escola Jatobazinho, Corumbá, Brazil | -18.5824 | -57.5144 | 340.87 | 580.36 |
| 250 | Fazenda Faro Moro, Paraguay | -21.7160 | -60.0148 | 282.75 | 343.97 |
| 251 | Fazenda Faro Moro, Paraguay | -21.7160 | -60.0148 | 428.29 | 698.17 |
| 252 | Fazenda Faro Moro, Paraguay | -21.7160 | -60.0148 | 251.70 | 410.18 |
| 254 | El Impenetrable National Park, Argentina | -25.1778 | -61.0924 | 182.93 | 121.14 |
| 256 | El Impenetrable National Park, Argentina | -25.1778 | -61.0924 | 230.99 | 848.22 |
| 257* | Captive-born  (Amazonian grandparents) | - | - | - | - |
| 258 | El Olimpo, Paraguay | -21.0472 | -57.8792 | 750.98 | 230.92 |
| Mean: | | | | 265.69 | 329.95 |

*For individuals 175, 225, 237 and 257, no coordinates of origin were included in the analysis, as they have no specific coordinates. However, the first three are from regions close to individuals 176, 224 and 236, respectively, so distances from these individuals were calculated. Individual 257 has grandparents with Amazonian origins, so we investigated whether the attribution is close to the Amazon region.

| 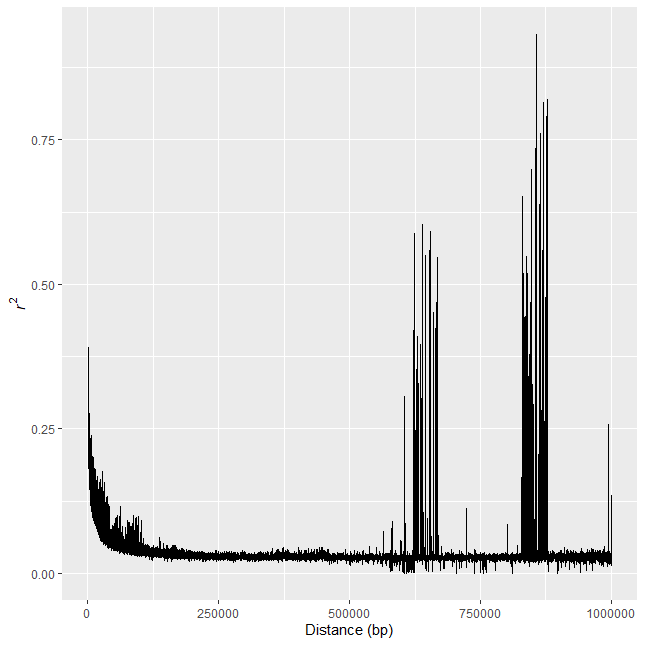 |
| --- |
| Figure S1 - Linkage disequilibrium decay against genetic distance (bp). Based on the plot, we removed one of a pair of SNPs if the LD value (r²) was greater than 0.1. |

| 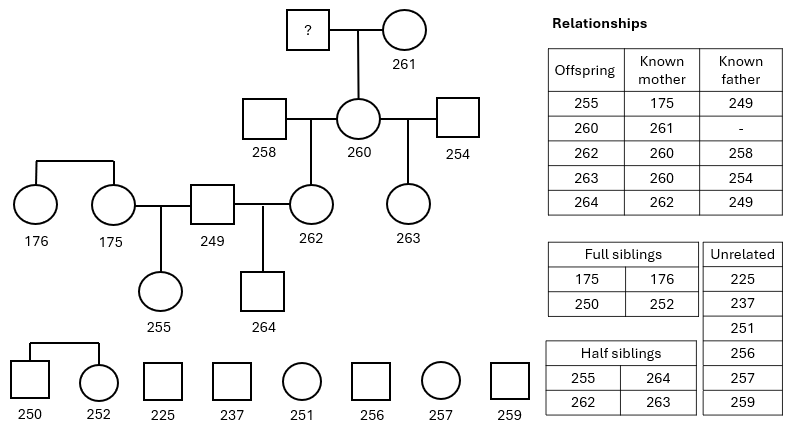 |
| --- |
| Figure S2 - Pedigree with the known relationships of the 18 novel whole-genome sequences reported here. Circles represent females and squares represent males. *Individual 176 had been previously sequenced as part of our original dataset and is reported by wildlife authorities to be the sister of 175. |
| 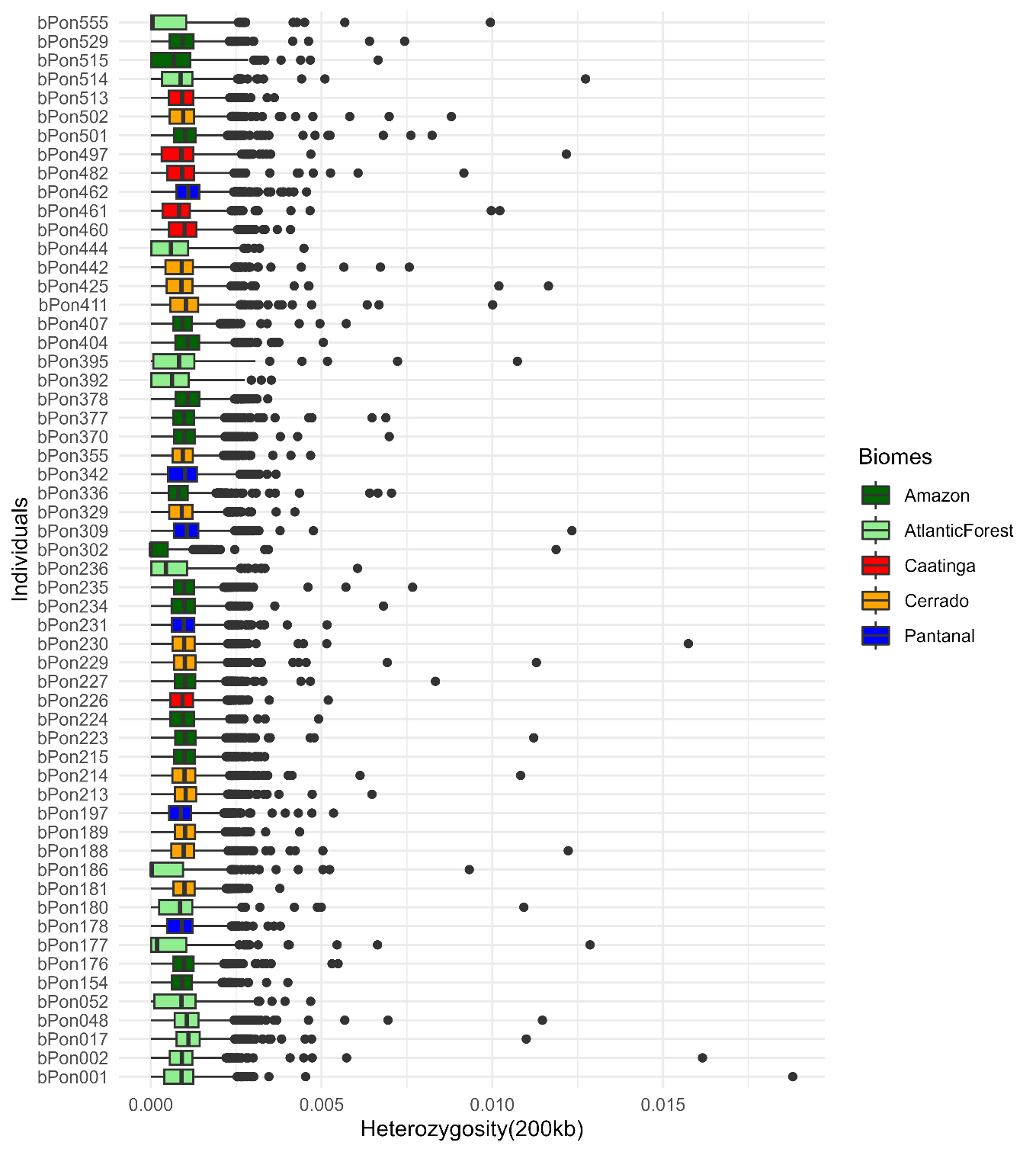 |
| Figure S3 - Heterozygosity levels by sample, using non-overlapping sliding windows of 200 kb. Box plots show 25th to 75th percentiles (boxes), medians (thick lines within boxes), and the maxima and minima (horizontal lines). |

| 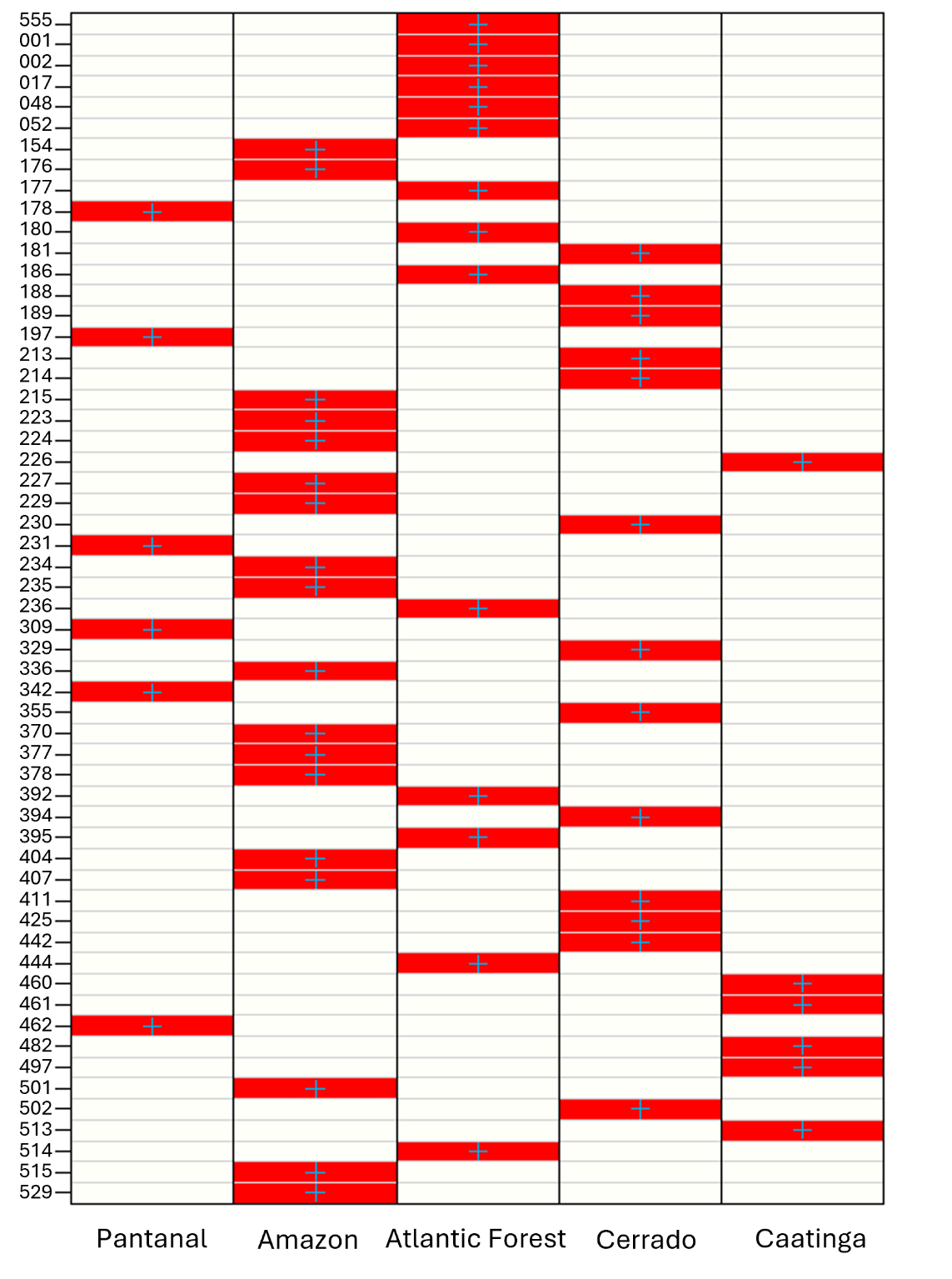 |
| --- |
| Figure S4 - Assignplot indicating the proportions of successful reassignment (based on the discriminant functions) of individuals to their original clusters, with the 84-SNP panel. Heat colors represent membership probabilities (red=1, white=0); blue crosses represent the prior cluster provided to DAPC. |

| 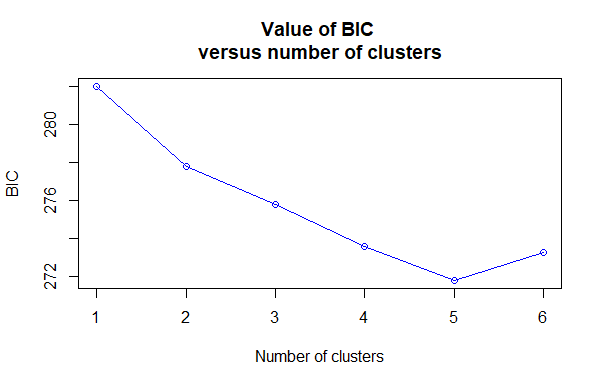  **(a)** | 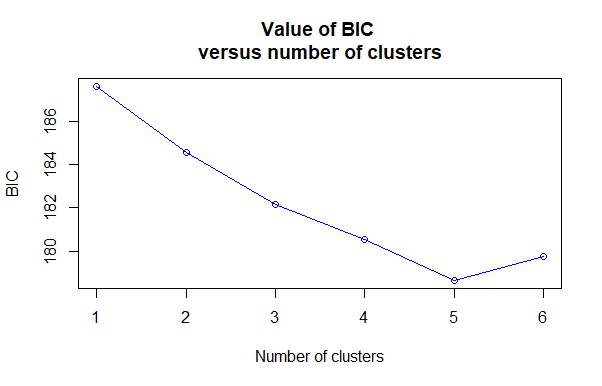  **(b)** |
| --- | --- |
| 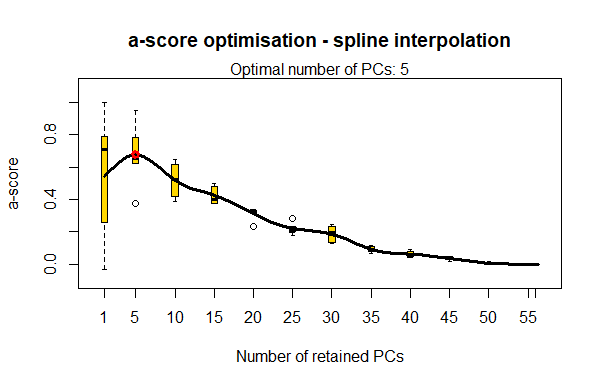  **(c))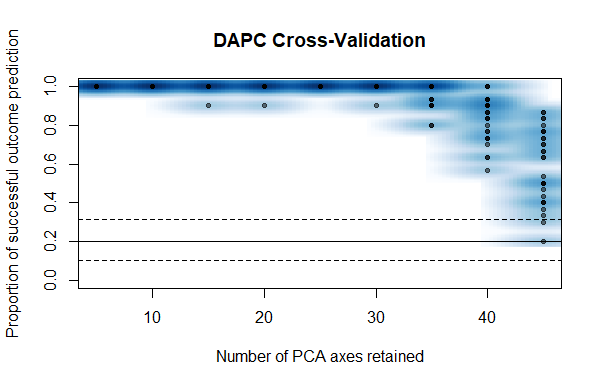)** | 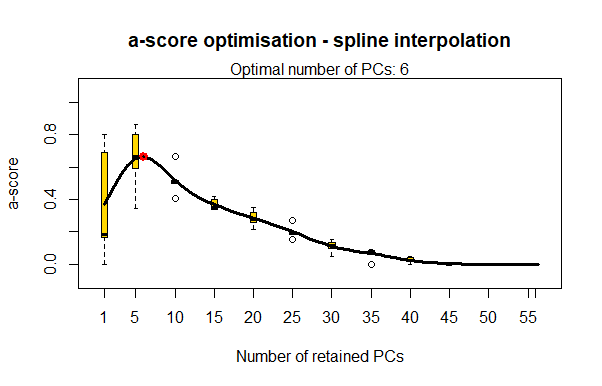  **(d))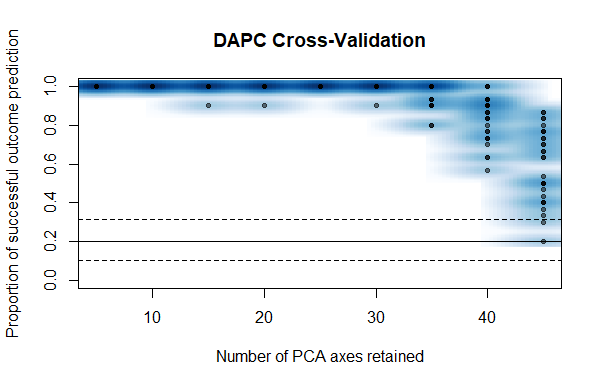)** |
| 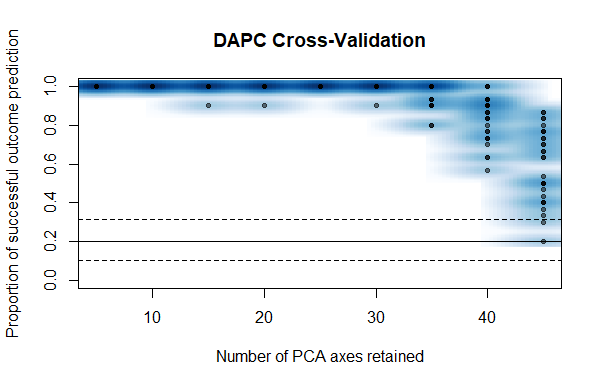  **(e))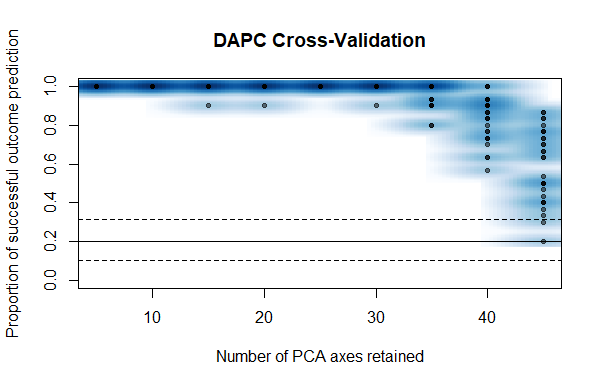)** | 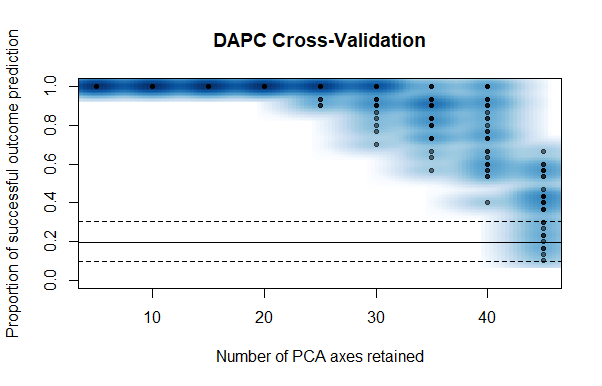  **(f))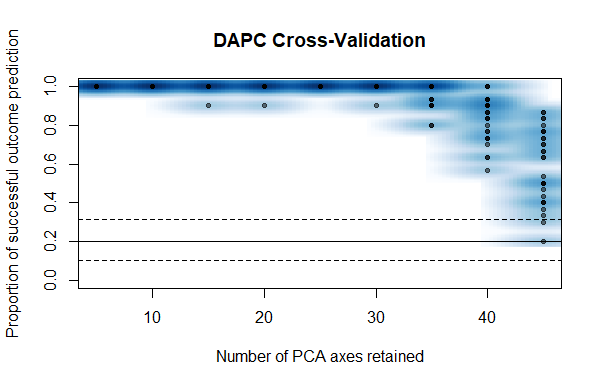)** |
| Figure S5 - Exploratory multivariate analyses conducted with ADEGENET v2.1.10, for the 459-SNP panel (459 SNPs; left) and its derivative (84 SNPs; right). (a-b) Graphs generated with *find.clusters()* showing a clear decrease of BIC until k = 5 clusters, after which BIC increases; (c-d) Procedure to define the optimal number of principal components (PCs), using the *optim.a.score()* function; (e-f) Cross-validation to define the number of PCs to retain based on proportion of sucessful outcome prediction, using *xvalDapc()* (training.set: 0,9; n.rep: 30). | |

| 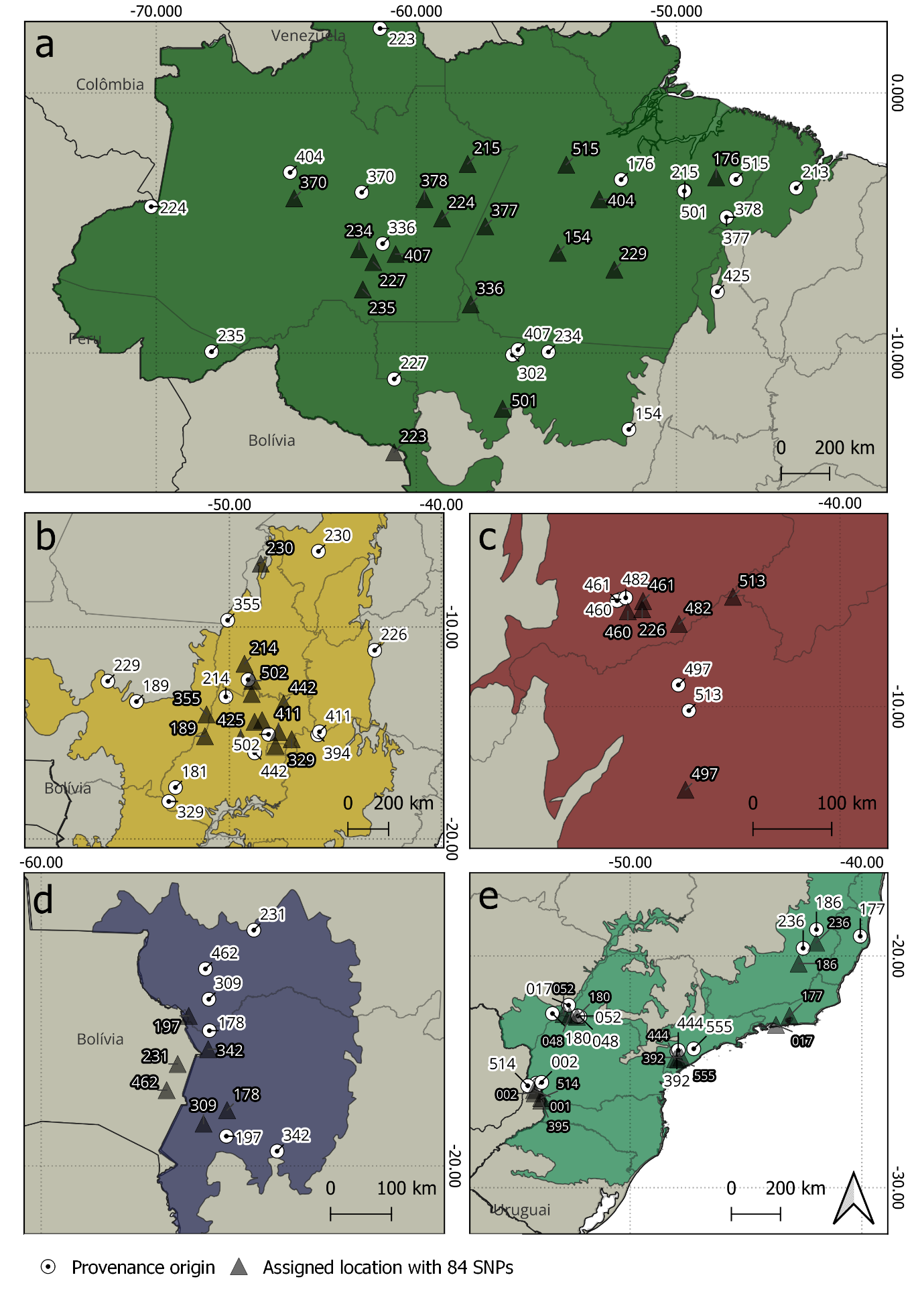 |
| --- |
| Figure S6: Geographic assignment with SCAT, using the 84-SNP panel. (a) Amazon; (b) Cerrado; (c) Caatinga; (d) Pantanal; and (e) Atlantic Forest. |

| 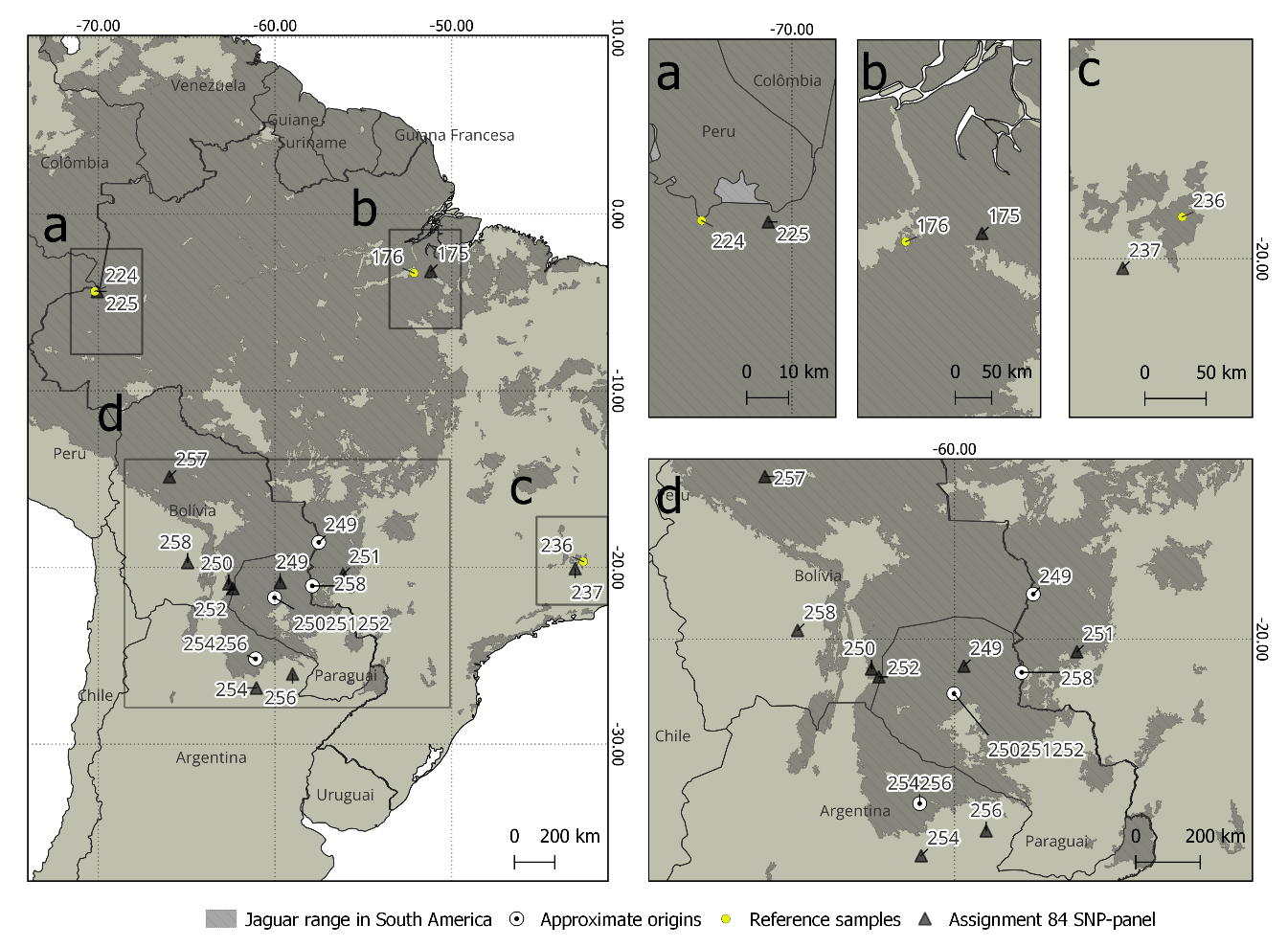 |
| --- |
| Figure S7 - Geographic assignment of validation samples with SCAT, using the 84-SNP panel and a reference dataset of 56 samples (broad view: left panel; close views: right panels). The triangles denote the assigned locations. The white circles denote the actual origins. The yellow circles denote samples from the reference dataset whose origins are close to the sampling locales for individuals 225 (a), 175 (b) and 237 (c). |

| 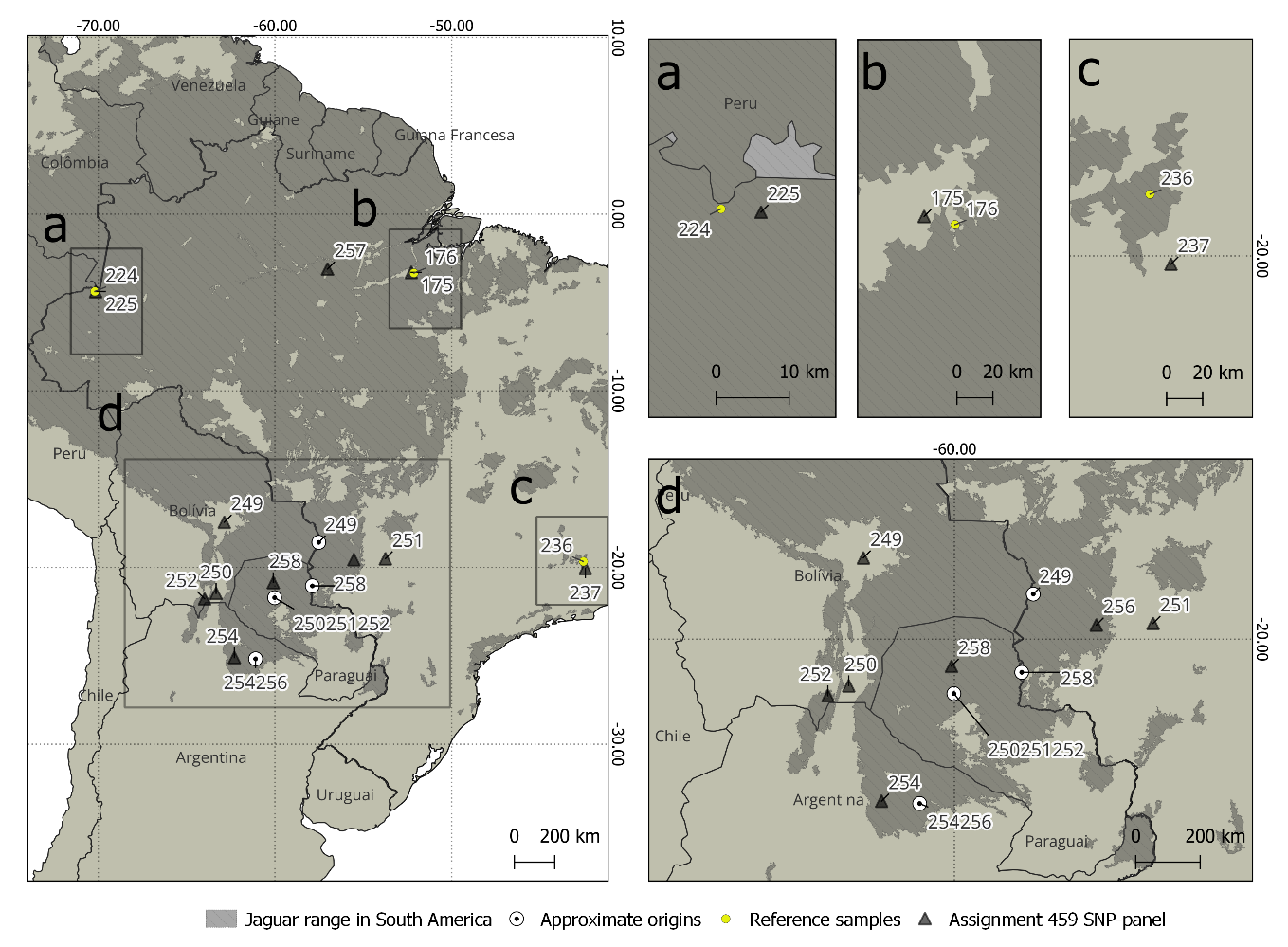 |
| --- |
| Figure S8 - Geographic assignment of validation samples achieved with SCAT, using the 459-SNP panel and a reference dataset of 56 samples (broad view: left panel; closer views: right panels). The triangles denote the assigned locations, while white circles denote the actual origins. The yellow circles represent samples from the reference data set whose provenance is very close to the collection locales for individuals 225 (a), 175 (b) and 237 (c). |
